# The Phylogeny of the Hymenopteran Brain

**DOI:** 10.1101/lm.053894.123

**Published:** 2024-05

**Authors:** Hans v. Alten

**Affiliations:** (From the Zoological Institute of the University of Freiburg i./Br. Germany)

For this, refer to 29 Figures in the text and 31 Figures on plates 18–21.

**Translation of the original publication by**

**Jürgen Rybak and Randolf Menzel**

**Content**. Introduction. Historical overview. Materials and methods. The physiology of mushroom body. The development of mushroom body. Specific part. **A.** Tenthredinidae. **B.** Cynipidae. **C.** Uroceridae. **D**. Ichneumonidae. **E**. Fossorians and Apidae. **a**) Fossorians and Archiapidae. **b**) Gastrilegidae and Podilegidae. **c**) Social Apidae. **d**) Parasitic Bees. **F**. Vespidae. General part. Summary and theoretical implication of the research results.

## Introduction

In some species of social Hymenoptera, a biologically fascinating fact has emerged that there is a sexual dimorphism in the structure of the central nervous system, apparently causally related to the different development of instincts.

After (Dujardin, 1850) first noticed a striking reduction in “substance corticale” in ant workers and emphasized the need to comparatively examine the brains of different sexes anatomically (Brandt, 1876), provided an overview drawing of the brains of a worker and drone of ***Apis mellifica*** and pointed out the existing differences, while (Forel, 1874, 1901) noted the same for Formicaries ants (Formicidae) and explained it with the help of schematic drawings.

More recently, following the start of my own research, a study by (Jonescu, 1909) has been published, in which the author conducts a detailed comparative examination of the three forms of bee brains (Translation Note (TN): worker (☿), drone (♂), queen (♀)) and identifies the differences between them.

It seemed interesting to me to delve deeper into the phylogenetic development of the Hymenopteran brain by studying the lower forms that have not been described or only insufficiently described so far, and to determine whether and to what extent differences exist between the central nervous systems of solitary and social Hymenoptera.

Furthermore, whether, as in social Apidae, sexual dimorphism already exists in solitary species or whether these differences emerged during the phylogenetic transition from solitary to social lifestyles.

At this point, I would like to express my sincerest gratitude to my esteemed teacher, Professor Weismann, for inspiring these investigations and for his consistently kind and enthusiastic interest in my work. I am also deeply thankful to Dr. W. Schleip, Assistant Professor (PD), for his valuable advice and providing materials.

## Historical overview

Following the works of older authors such as (Swammerdam, 1737-1738), (Cuvier, 1809-1810), and (Treviranus, 1802), (Dujardin, 1850) resumed the study of the brain of ***Apis mellifica*** and provided the first detailed information about it. Fourteen years later, (Leydig, 1864) expanded on the research of his predecessors and extended it to other Hymenoptera (ants, wasps) for comparative purposes. While there is little to add regarding the external shape based on his accurate illustrations and descriptions, Leydig himself considered the knowledge of the internal structure and organization of the arthropod brain as “a vast, undeveloped field.” Since then, many authors have worked in this direction, including (Rabl-Rückhard, 1875), (Dietl, 1876), (Berger, 1878), (Flögel, 1878), followed by more recent researchers like (Viallanes, 1882), (Cuccati, 1887-1888), and last (Kenyon, 1896a) with his classic work “The brain of the bee,” and more recently (Haller, 1905), (Janet, 1899), and (Jonescu, 1909).Thanks to improved cutting and staining techniques, these authors have provided us with a precise understanding of the anatomy of the arthropod brain, specifically the brain of social Hymenoptera.

Haller and Jonescu, at the beginning of their works, compiled and compared the known facts so extensively that I can refer to their contributions. At this point, I would like to present a table of the individual parts of the brain of ***Apis mellifica*** (according to (Jonescu, 1909)) along with their names and synonyms. A detailed discussion of specific parts that are particularly relevant to this study will be reserved for later chapters.

**We distinguish:**

I. The supraesophageal ganglion (le cerveau (Viallanes, 1882) dorsocerebrum (Kenyon, 1896a)
  A. **Protocerebrum**
    - The protocerebral lobes (Les lobes protocérébraux,(Viallanes, 1882) Fig. 4,16 on Plate 18/19 L.pr
    - Pilzhutförmige Körper (Les corps pédonculés (Dujardin, 1850), Mushroom bodies (Kenyon, 1896a) Fig. 5 on Plate 18 PK
    - The central body (Fan-shaped structure (Dietl, 1876) Fig. 19ff. on Plate 20 CK
    - The nuclei of the central body (les tubercules du corps central, (Viallanes, 1882) ocellar glomeruli (Kenyon, 1896a) Fig. 17 on Plate 20 Gl.oc
    - The intercerebral bridge (ocellar nerve bridge (Jonescu, 1909); le pont des lobes protocérébraux (Viallanes, 1882) fibrillar arch, (Kenyon, 1896a) TextFig. 4, Fig. 14 on Plate 19 Po-int
    - The ocellar nerves Fig. 20 on Plate 20 N.oc
    - Lobus opticus - Sehlappen (Leydig, 1864) Fig. 1–6 on Plate 18 L.opt
    1. The subretinal nerve bundle layer (la couche des fibres post-rétiniennes (Viallanes, 1882) Fig. 7 on Plate 19 SNbs
    2. The outer fibrillar mass Fig. 7 on Plate 19 M.m.e
    3. The outer crossing Fig. 11 on Plate 19 Ch.e
    4. The middle fibrillar mass Fig. 7 on Plate 19 M.m.m
    5. The middle crossing Fig. 11 on Plate 19 Ch.m
    6. The inner fibrillar mass Fig. 7 on Plate 19 M.m. i
    7. The inner crossing Fig. 7 on Plate 19 Ch.i
  B. **Deutocerebrum**
    - The lobi olfactorii (Riechlappen (Leydig, 1864) lobe olfactive (Viallanes, 1882) Fig. 1–6 on Plate 18, Fig. 16 on Plate 19 L.olf
    - The sensory antennal nerves I and II
    - The motor nerves of the antenna
  C. **Tritocerebrum**
    (Viallanes, 1882) The labrofrontal nerve
    (Haller, 1905) The inner motor nerve of the antenna
    (Janet, 1899) Le nerf du muscle dilatateur inférieur du pharynx
II. The subesophageal ganglion (ventrocerebrum (Kenyon, 1896a)
    - Connectives of the supraesophageal with the subesophageal ganglion TextFig. 8b C.oe
    - Mandibular ganglion
    - Maxillary ganglion
    - Labial ganglion

TN: The Figures on Plates 18–21 can be found in supplementary file S1: on Plates 18–21.

## Materials and Methods

A portion of the processed solitary Apidae, supplied to the local Zoological Institute by Dr. Friese-Schwerin, was kindly made available to me by Professor Weismann. I personally collected another portion, as well as many Vespidae and Bombus species, during the summer of 1909 in the Black Forest and on the sunny loess slopes of the Kaiserstuhl. The Tenthredinidae, in a well-preserved state, were provided to me by Teacher P. Eigen-Solingen.

A portion of the material was preserved in alcohol, where the general size and positional relationships of the parts of the cerebrum could still be clearly identified, but finer structural details could not always be adequately studied.

In some cases, freshly captured animals were treated with the Henning's mixture (Hennings, 1900, 1904). However, much better results were obtained using the hot Gilson mixture in the modification suggested by (Petrunkewitsch, 1901).

The most suitable method proved to be a solution of approximately 9% formaldehyde, into which the freshly excised brains or whole animals were placed after the mouthparts were cut at the base with scissors or the cranial cavity was opened by removing the anterior chitinous cover.

In most cases, the brains were dissected before or after fixation and sectioned into serial slices, including frontal sections (parallel to the anterior surface of the head), sagittal sections (in the direction of the sagittal plane), and horizontal sections (perpendicular to the frontal and median planes). For staining the specimens, I generally used hematoxylin (Delafield & Prudden, 1904) ^1^ with counterstaining by acidified eosin or picric carmine. For the specific study of fiber pathways, picronigrosin (TN: a solution of nigrosin in picric acid, used for staining connective tissue*)* was used after sublimate fixation, while a copper sulfate impregnation followed by staining with hematoxylin-Weigert or hematoxylin-Kenyon was applied after formaldehyde fixation (see (Kenyon, 1896a)). The solution consists of 1 cc of 10% phosphomolybdic acid, 1 g of hematoxylin crystals, 6 to 10 g of chloral hydrate, and 100 cc of distilled water). This method yields excellent results, although the overall view may be slightly hindered by the uniformity of staining. Total preparations were treated with borax carmine.

### The physiology of the mushroom body

As we will primarily focus on the mushroom body in the subsequent discussion, I would like to briefly address their significance.

(Dujardin, 1850), who first discovered them and referred to them as “lobes à convolutions” based on their appearance in transparent total preparations, compared them to the convolutions of the human cerebrum and considered them as “organs of intelligence.” However, this view did not go unchallenged (Leydig, 1864), who described the mushroom body as “stalked bodies,” considered it not unlikely that their development would go hand in hand with the increasing formation and enhancement of visual capabilities. On the other hand, (Rabl-Rückhard, 1875) raised the objection that they were well-developed even in a blind African ant, Typhlopone (***Dorylus fulvus***, army ants), and therefore cannot be directly associated with vision. (According to (Haller, 1905), the development of the mushroom body even has an inverse relationship with the development of the optic lobe). (Dietl, 1876) does not lean towards the view of an intellectual function, although he found these organs to be more developed in Hymenoptera compared to Orthoptera. In contrast, (Forel, 1901), supported by (Brandt, 1876), shares Dujardin's perception and relies on his findings regarding the different development of these structures in ant males, females, and workers. (Flögel, 1878), who established the forms with less developed and more prominent mushroom body through extensive comparative anatomical investigations, provides valuable evidence to support this view. Finally, (Jonescu, 1909) work demonstrates that there are differences among the three forms of bees based on the distinct development of instincts, and these differences primarily manifest in the size and shape of the mushroom body.

Attempts were also made to resolve the undecided question through physiological experiments. (Faivre, 1857) found that the ventrocerebrum is responsible for coordinating the muscle movements of the body, to which (Binet, 1894) declared the dorsocerebrum as “the seat of power that directs these movements.” According to (Binet, 1894), a brainless insect behaves similarly to a vertebrate deprived of its cerebral hemispheres (such as a pigeon).

It can still live for months, eat when the food is placed directly between its palps, but is unable to approach the food, even if it is only a short distance away. However, Kenyon points out that these results are not entirely conclusive, as neither olfactory nor visual stimuli could be transformed into motor impulses in the mentioned experiments. Whether the insect would behave the same if the lobus opticus and lobus olfactorius were left intact and only the mushroom body were destroyed remains a question that has not been answered yet, and its solution would likely involve significant technical difficulties.

The purely anatomical investigation has also yielded quite interesting results. (Kenyon, 1896b) using his bichromate-silver method, found that the ganglion cells of the bee's mushroom body have a distinctly different shape compared to all other ganglion cells. They bear some resemblance to the shape of Purkinje cells in higher vertebrates. Each cell in the calyces of the mushroom body (**TextFig. 1A** and **B**) sends out a finely branching dendrite (d) into the neuropil of the calyx wall. Just before entering, it gives off a fine branch, the neurite (n), which moves along the wall of the calyx towards the stalk (St), where it extends to the origin of the outer (ra) and inner root (ri), where it then dichotomously divides (bifurcation of the stalks) and sends a branch into each root. These “intellective cells” are characteristic of the bee's brain according to Kenyon and are not found, for example, in higher crustaceans, so the ganglion cell accumulations found there cannot be homologized with the mushroom body of the Apidae.

**TextFig. 1A:**
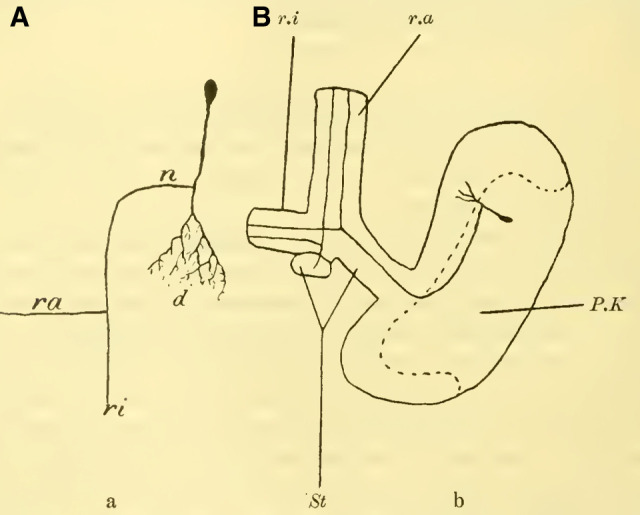
“Intellective cell” of the mushroom body of ***Apis mellifica.* B**: Mushroom body (P.K) of the right side from above. The outer P.K is shown in cross-section, the inner one is cut off. After Kenyon, 1896. d Dendrite, n Neurite, r.a anterior root, r.i inner root, P.K mushroom body, St stalk

In addition to these “intellective cells” (Kenyon, 1896a) distinguishes five other elements in the bee's brain:

**1** Afferent or sensory fibers
**2** Connecting fibers
**3** Efferent fibers
**4** Commissural fibers
**5** Association fibers

Through manifold fiber tracts and commissures, the mushroom bodies are connected to all important centers of the brain. In some cases, the association fibers allow an invading sensitive stimulus to jump over immediately to a motor cell, creating a connection analogous to the reflex arc of higher animals. The rest allow the stimulus to take a direct (reflex) or indirect path, namely, first to the cells of the mushroom bodies and from there through the fibers of one or more association cells to the motor cells. From these considerations, Kenyon concludes that the cells of the mushroom body, “so distinguished by their shape and position, are the elements which control or produce the actions that are called intelligent” (Kenyon, 1896a).

On the basis of these physiological, anatomical and comparatively anatomical, as well as on the basis of the following findings, as I would like to note right here, one will probably not to go too far if one addresses the mushroom bodies as the main reflex and association centers of the hymenopteran brain. They could also be described as “organs of intelligence” if one assumes that bees possess psychic abilities in the sense of (Bethe, 1897b) (sentience, ability to form new associations on the basis of memory and experience), which is as vividly disputed, (Bethe, 1897b)) on the one hand as it is claimed on the other ((Buttel-Reepen, 1900, 1903) (Forel, 1874, 1901) (Wasmann, 1901, 1909)).

The formation of the syncerebrum can now be influenced by manifold moments. The external shape e.g. (Viallanes, 1882) by the type of food, in that a wide esophagus causes an elongation of the pharyngeal commissures and keeps the parts of the subesophageal ganglion more apart (*Blatta*; Coleoptera) than a narrow one (Hymenoptera, Diptera). This prolongation of the pharyngeal commissures can go so far that the subesophageal ganglion comes to rest in the prothorax (*Rhizotrogus* (Scarabaeidae) (Binet, 1894). Furthermore, the mere shape of the head can also be significant. The most far-reaching changes, however, will be experienced by the parts of the brain which, with the increasing development of the sensory organs, are destined to transmit and process the stimuli transmitted by them. Thus, a better vision will affect the development of the optic lobe (dragonflies), the olfactory lobe, and finally an increase and complication of instincts will affect the development of the mushroom bodies.

Accordingly, when we compare a relatively closed group like the Hymenoptera, we will expect to find the most striking differences in the three parts of the brain mentioned, which is why they can perhaps be contrasted with a certain restriction as variable parts as constant parts, similar to what Jonescu proposes for the three forms of ***Apis mellifica***. We will therefore primarily consider the development of these three variable parts.

### The development of the mushroom body

The lowest forms of the mushroom body in arthropods, which would be considered as simple differentiations in the ganglion cell cortex of the protocerebrum, are no longer present. Even in the lowest forms of tracheates, such as onychophorans and myriapods, they already show such a degree of development that we can no longer consider them as primitive (Haller, 1905). Lower states similar to myriapods can be found in branchiopods (Leydig, 1864), but the globuli are shifted so far backward that they even touch the antennal ganglion in ***Porcellio scaber*** (woodlouse, Isopod, Crustacea) (Haller, 1905) and this backward displacement also persists in decapods (Bellonci, 1883), (Krieger, 1880), (Bethe, 1897a, 1897b) (Bethe, 1897a). Furthermore, (Berger, 1878) found no evidence of differentiation in the entomostracans (*Artemia*, phyllopod, branchiopods), so it is undoubtedly necessary to assume not only a convergent development but also a diphyletic origin of the mushroom body for the two subphyla of branchiates and tracheates, similar to what is assumed for the compound eye.

The simplest conditions for tracheates are found, as expected, in onychophorans. The ganglion cell cortex of the brain of ***Peripatus capensis*** (velvet worm, Onychophora) (Balfour, 1883), (Saint-Remy, 1890) is almost entirely composed of similar, protoplasm-poor elements, while cells with relatively abundant protoplasm are scarce and scattered. Additionally, these protoplasm-poor cells are more closely arranged and more strongly stained with dyes than those in the surrounding regions, specifically in the middle region of the cerebrum, directed forward and somewhat ventrally. These cell masses (masses ganglionnaires antérieures, (Saint-Remy, 1890)) are homologous to the globuli^2^ or mushroom body cells in higher organized tracheates. The processes of these unified cell masses form two bundles of fibrils on each side, which apparently merge within the globulus and form a “stalk” that extends to the so-called “medullary mass” (TN**:** neuropil). The neuropil is connected to the rest of the ganglion cell mass and all other parts of the brain, likely including the neuropil and stalk of the opposite side, through numerous processes.

Thus, in ***Peripatus capensis*** (velvet worm), while only paired differentiations of the cerebral cortex are present on each side, the paired bundles of fibrils within each globulus indicate a later separation, which is already observed in myriapods.

Among myriapods, (Haller, 1905) found simpler conditions in chilopods than in diplopods. His illustration of the brain of the millipede ***Julus terrestris*** (diplopode, Myriapoda) clearly shows that the globuli have shifted from the anterior ventral surface of the cerebrum to the dorsal part, and a beginning constriction can be observed at the edge of each globulus, although it does not lead to complete division. From the posterior and lateral parts of each globulus, a stalk initially descends towards the midline and unites there, after giving off a commissure, with the one from the opposite side. Separately, fibers from the medial parts of the globuli gather, also connected by a direct commissure, then take a downward course, located laterally and behind the shared first stalks, and eventually unite ventrally and bend forward, ending beneath the anterior ganglion cell layer of the brain (cf. on **Plate 12** and Figs. 4, 5 from (Haller, 1905)).

From this point onwards, even if we assume a monophyletic origin of the mushroom body in tracheates, their further development in the various orders of hexapods must be considered as separate and potentially convergent, as different stages of development can be found among the suborders and families of almost all insect orders.

For example, according to (Flögel, 1878), in some small butterflies, there is only one globulus on each side with a barely recognizable wall neuropil, while in *Cossus* (goat moth, Cossidae), *Sphinx* (hawk moth, Nymphalidae), and *Vanessa* (noble butterflies, Nymphalidae), four globuli with well-developed, albeit small, calyces occur. In lower orthopterans (***Forficula***, earwigs, Dermaptera), ***Acridium*** (short-horn grasshopper, Acrididae), there are only two globuli, and their neuropil still forms a simple, broad plate, while in ***Acheta*** (cricket, Orthoptera) and ***Gryllotalpa*** (mole cricket), a budding division is already underway ((Dietl, 1876), which is completed in ***Blatta*** (cockroaches, Blattodea) (Newton, 1879), (Haller, 1905), where the mushroom body develop to a similar extent as in hymenopterans.

Similarly, Haller found in a coleopteran, ***Procrustes coriaceus*** (today: ***Carabus coriaceus***, ground beetle, Carabidae), that the cell layer of the globuli is not yet differentiated from the surrounding tissue, although there are already two stalks on each side, whereas in ***Dytiscus*** (diving beetle, Dytiscidae) (Berger, 1878)) and ***Hydrophilus*** (water scavenger beetle, Hydrophilidae) (Flögel, 1878), the ganglion cells of the globuli are clearly distinct from the adjacent cells and have separated into two parts on each side.

However, while we assume independent and convergent development in each order, it is not excluded that the forms observed in individual species can also be considered as a result of regression.

### A. Tenthrediniden (True sawflies)

The species investigated were: - ***Tenthredo mesomelaena - Tenthredo flava - Allanthus scrophulariae - Allanthus arcuatus - Encarsioneura Sturmii***

I would like to note at the outset that, except for absolute differences in size, no noticeable variations were observed among the species investigated, so the following description should have general applicability to the studied forms.

Regarding the brain of sawflies (Tenthredinidae), I am only aware of a brief note by (Flögel, 1878) who observed “rudimentary calyces” in *Tenthredo ribis*, similar to those found in Neuroptera and Coleoptera.

**Figure 1 (on Plate 18)** shows a frontal view of the brain of ***Tenthredo flava*** (***Hoplocampa flava***, Tenthredinidae) after complete dissection and alcohol preservation. In the middle, we can observe the main mass of the syncephalon, from which the olfactory lobes (Lobi olfactorii) protrude forward and slightly ventrally, extending into the antennal nerves (Na). The anterior surface shows a very shallow, barely discernible medial groove, which becomes noticeable as a small indentation at the upper dorsal edge of the brain (“le sillon cérébral médian,” (Viallanes, 1882). No further external differentiation is readily apparent, especially no dorsal bulge that would indicate the presence of mushroom body. Adjacent to this central part, each side is sharply demarcated by the voluminous, well-developed optic lobe (Lobus opticus) with the retina (Ret) extending onto the anterior surface.

Further knowledge of the internal structure of the brain is obtained through sections, revealing the essential components of the brain of social Hymenoptera, albeit with varying degrees of development.

The globuli are observed as unpaired differentiations on each side in the ganglionic cell layer of the brain. A division is indicated by a slight indentation (**TextFig. 2**, Sp) at the posterior edge, but it is not fully developed, which is why the furrow (la scissure du corps pédonculé, (Viallanes, 1882)) is not clearly pronounced. This furrow, which is particularly well-defined in the highest-ranking Hymenoptera (*Vespa*), forms the externally visible boundary between the ganglionic cells belonging to the inner calyx neuropil and those belonging to the outer calyx neuropil of the mushroom body. This boundary also exists in Tenthredinidae and undoubtedly runs through the indentation located on the back of the globuli between the two masses, from posterior to medial and from anterior to lateral. Despite the incomplete separation, for the sake of convenience, I refer to an external and internal globulus (comparable to Uroceridae and Cynipidae).

**TextFig. 2.**
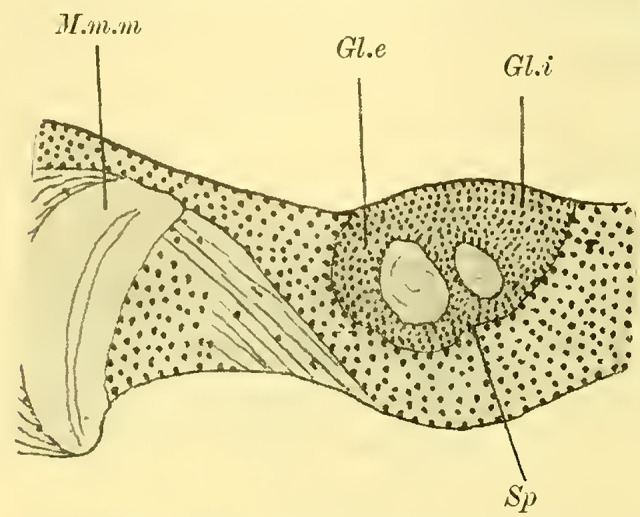
Horizontal section through the left half of the brain of ***Allanthus scrophulariae.*** Gl.i Internal globulus, Gl.e External globulus, M.m.m: medulla of the lobus opticus, Sp Indication of the furrow. Scale: 1:80.

The globuli are located on the dorsal surface of the protocerebrum, not touching those on the opposite side in the midline (see also **Fig. 7 on Plate 19**). They slightly extend onto the posterior surface, with the inner globulus (**TextFig. 2** Gl.i) slightly shifted forward compared to the outer posterior globulus (Gl.e). The cells of the globuli are clearly demarcated from the adjacent cells of the pars intercerebralis (Haller, 1905) (**Fig. 7** P.i, **on Plate 19**) and are also conspicuously different from the cells of the optic ganglia in terms of their size, arrangement, and nucleus-to-protoplasm ratio. **Fig. 8a–c (on Plate 19)** illustrates these relationships, depicting the three cell types at the same magnification. **Fig. 8a (on Plate 19)** shows one of the cells of the pars intercerebralis, which are otherwise not uniformly sized. The large pear-shaped cell contains abundant finely granulated protoplasm, with the nucleus occupying about 1/8 of the cell volume and set apart by a bright halo. The chromatin is partly distributed in fine granules on the meshwork of the nuclear framework, while most of it is concentrated in the highly stainable nucleolus.

The cells of the lobus opticus (**Fig. 8b, on Plate 19**) are significantly smaller and irregular in their outer form, but they are approximately the same size as each other. They are closely packed together within the fine neuroglial network that surrounds them and are connected by protoplasmic bridges, as observed in ***Blatta*** (cockroach) according to (Haller, 1905). I could not clearly demonstrate such protoplasmic bridges in the Tenthredinidae, but I do not immediately deny their existence. These cells have less protoplasm compared to the elements of the pars intercerebralis, but their nucleus is relatively larger, occupying about 1/4 of the total cell volume. The chromatin is distributed in fine granules, while a nucleolus is absent.

The cells of the globuli are even smaller (**Fig. 8c, on Plate 19**), and they are uniform in size among themselves and closely packed together. The ratio of nucleus to protoplasm shifts further in favor of the nucleus, which nearly fills the entire cell. The nucleus contains abundant, scattered chromatin in granular form and is known to be highly stainable. These cells send out processes to neighboring cells, with some of them terminating in very fine, thread-like neurites. On each side, these neurites gather into two extensive fiber bundles known as the stalks. These stalks, which serve as the neuropil of the globuli, do not yet exhibit the cup shaped calyces found in higher Apidae species. They do not even possess the form of a calyx-shell but only show bulb-like swellings at their ends (**TextFig. 3,** St).

**TextFig. 3.**
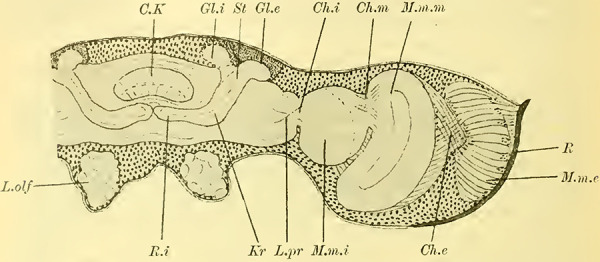
Section through the brain of ***Tenthredo flava*** ♀. Plane between the horizontal and frontal planes. Semi-schematic (the indicated internal globulus is not captured in the preparation). R Retina, M.m.e lamina M.m.m medulla, M.m.i lobula, Ch.e, Ch.m, Ch.i external, middle, and inner chiasma, St Stalk, Kr bifurcation of stalks, R.i Inner root, L.pr Lobus protocerebralis, Gl.i Internal globulus, Gl.e External globulus, CK Central body, L.olf Lobus olfactorius. Scale: 1:64

The stalks soon align closely together within the surrounding mass of globuli cells (**Fig. 7** St, **on Plate 19**) and follow a peculiar course. Initially, they descend downward to the lowest point, which lies below the central body. Along this path, the stalks first slightly bend backward, forming a gentle curve with the concavity facing forward and upward (sagittal section, **Fig. 9** St, **on Plate 19**). They then form another curve with the concavity directed medially. At the lowest point, the bifurcation of the stalks takes place (**Fig. 11**, Kr, **on Plate 19**), where their fibers interpenetrate and divide dichotomously, with one branch entering the anterior root originating here and the other entering the inner root, as mentioned earlier.

The anterior root then extends directly forward, slightly upward (**Fig. 10** R.a, **on Plate 19**), and laterally (**Fig. 11** R.a, horizontal section, **on Plate 19**), bending and ending in a club-shaped swelling beneath the ganglionic cell layer on the front side of the brain, while the inner root (**TextFig. 3;** **Fig. 7**, 11 R.i, **on Plate 19**) runs slightly backward and then ascends, meeting the root coming from the other side below the central body (CK), without, however, merging with it (‘Balkennaht’ = beam seam of older authors).

These peculiar conditions make it impossible to trace the entire course of the stalks, even with the exclusion of the anterior roots, on a frontal section. Therefore, **TextFig. 3** of a section taken from back-top to front-bottom direction is somewhat schematic, as the depicted inner globulus is no longer encountered. In **Fig. 7, on Plate 19**, the left stalk is cut three times.

#### Lobus opticus

(Haller, 1905) has stated that the development of the mushroom body is in inverse proportion to that of the lobus opticus. While this statement cannot be universally applied, exceptions can be observed in individual cases. In Tenthredinidae, in contrast to the limited development of the MB globuli, the lobus opticus is well-developed in all its typical parts (**Fig. 7, 11, on Plate 19**).

Following the outermost retina (Ret), there is the subretinal nerve bundle layer (S.Nbs, la couche des fibres post-rétiniennes, (Viallanes, 1882)), whose fibers lead to the outer fibrillar mass (M.m.e., TN: from here on lamina). This lamina, resembling a concave-convex lens with its concavity directed medially, corresponds in size and shape to the facets of the compound eye and exhibits similar structural relationships as in ***Apis mellifica***. At the outermost layer, there is a layer of ganglion cells (la couche à noyaux, (Viallanes, 1882) **Fig. 7, 11a, on Plate 19**), followed by a layer of fibrillar neuropil (la couche des palissades, (Viallanes, 1882); spindle layer, (Jonescu, 1909)), and the third layer consists of small weakly stained cells and a fibrillar network, which can be further divided into three parts. Two outers fine fibrillar networks are formed by the processes of the ganglion cells on both sides (**Fig. 7, 11b, on Plate 19**), enclosing a thicker middle fibrillar network. Regarding the finer structure of the spindles and the relationship of the first layer of ganglion cells (a) to these and the subretinal nerve bundle fibers, I refer to (Jonescu, 1909); it is the same as in ***Apis mellifica***. The nerve fibers emanating from the lamina pass through the outer chiasma (Chiasma externum, **Fig. 7, 11,** Ch. e, **on Plate 19**) to the middle fibrillar mass (Massa medullaris media, M.m.m TN: from here on medulla). The fibers of this outer chiasma, which can only be well-traced in sagittal and horizontal sections, are composed to a lesser extent of the neurites of the cells a of the lamina (Kenyon, 1897), as they pass through the spindle layer and penetrate directly into the medulla through the chiasma. The majority is formed by the neurites of the ganglion cells around the medulla (Haller, 1905), (Parker, 1897) and the outer chiasma (Jonescu, 1909). These neurites penetrate distally into the neuropil.

The medulla (M.m.m), which also possesses the shape of a concave-convex lens, faces laterally with its concave surface and medially with its convex surface in Tenthredinidae (**Fig. 11 on Plate 19**). The predominantly radial, partly tangential course of the fibers determines the peculiar structure of the neuropil, where two dark outer regions and one bright middle region can be distinguished.

The middle chiasma (Chiasma medium, Ch.m), which is particularly clear in horizontal sections, connects the medulla with the inner medullary mass (TN: from here on: lobula). Additionally, the medulla is directly connected to the lobi protocerebrali. First, through the Fasciculus superior anterior (F.s.a.: **Fig. 16 on Plate 19, Fig. 18 on Plate 20**). This fiber connection, as well as all subsequent ones, also exists in Tenthredinidae. However, since they are not always captured in the presented sections, in such cases, I refer to other Hymenopteran sections where they have been depicted for orientation. It originates from the upper anterior to middle margin of the medulla; a portion of its fibers penetrates directly into the calyx neuropil of the globuli (cf. **Fig. 23 on Plate 20**), while another portion extends downward to the “Fasciculus optico-antennalis” and crosses paths with a small bundle coming from the lower margin of the medulla (lower part of the fasciculus superior anterior, cf. **Fig. 26** *UT* **on Plate 20**), with some of its fibers entering the calyces and others extending to the Fasciculus optico-antennalis (Jonescu, 1909)). The middle part of the Fasciculus superior anterior immediately gives rise to the Fasciculus superior posterior (Faisceau supéro-postérieur, (Viallanes, 1882); cf. **Fig. 18,** F.s.p, **on Plate 20**), which connects with its counterpart from the opposite side through the commissures, thus establishing a direct connection between the medullae of both optical lobes. Other fibers of the Fasciculus superior anterior enter the protocerebrum and cross through the anterior commissure (**Fig. 11** C.a, **on Plate 19**) to the other side. Finally, from the origin of the Fasciculus superior posterior, a direct small bundle (Faisceau anastomotique, (Viallanes, 1882) extends to the lobula.

The lobula (**Fig. 7, 11,** M.m.i, **on Plate 19**) appears circular in frontal sections and more oval in horizontal sections. Structurally, it can be divided into a distal radially striped part and a proximal more homogeneous-looking part. According to Jonescu's findings on pupae of ***Apis mellifica***, (Jonescu, 1909) this inner part of the lobula is thought to be formed by the fusion of two lenses, an outer concave-convex lens and an inner more biconvex lens, which allows for its homologization with the lobus opticus of crustaceans. According to this hypothesis, the lamina of Hymenoptera corresponds to the “first optic ganglion” (Parker, 1897), of crustaceans, the outer chiasma to the “first decussation,” the medulla to the “second optic ganglion,” the middle chiasma to the “second decussation,” the outer (radially striped) part of the lobula to the “third optic ganglion,” and the inner part of the lobula to the “fourth optic ganglion.”

As compelling as this hypothesis may initially seem, I believe that there are significant objections to it, particularly regarding ontogenesis. Indeed, when examining pupae, it appears that the lobula consists of two parts, but a similar situation can also be observed in the medulla. However, in both cases, the tangential course of the fibers within the neuropils, emitted from the posterior medial edge of the lobula and the anterior medial edge of the lamina by the fasciculus inferior posterior or the fasciculus superior anterior and posterior, respectively, creates the impression that each of the laminas consists of two separate lenses.

Furthermore, there is no evidence from phylogeny to support the aforementioned hypothesis. In lower Hymenoptera (Tenthredinidae) as well as in lower tracheates, the presence of four neuropils in the lobus opticus is not observed. Although there are certain similarities in the structure of the lobus opticus in lower tracheates (myriapods, (Haller, 1905) and branchiates (***Artemia***, (Berger, 1878), these similarities exist before the differentiation of the typical neuropils. Their development must have occurred independently in both lineages, just like the globuli and the compound eye, but in this case, it led to non-congruent results, with four neuropils in crustaceans and at most three in tracheates.

Moreover, the fibers between the third and fourth neuropils in crustaceans form a chiasma (third decussation), while in Hymenoptera, the fibers of the middle chiasma, which enter the posterior medial edge of the lobula, pass straight through both the radially striped part and the more homogeneous part, as emphasized by Jonescu in his otherwise very detailed description.

For all these reasons, I believe that a homologization of the parts of the lobus opticus in crustaceans and Hymenoptera is not feasible.

From the lobula, the fibers enter the lobus protocerebrales, partially crossing over, justifying the proposed name “inner chiasma” by (Jonescu, 1909) (cf. **Fig. 7,** Ch.i, **on Plate 19**). These fibers can be grouped into two major bundles: the infero-anterior fascicle (**Fig. 11,** F.i.a. **on Plate 19**; faisceau inféro-antérieur, (Viallanes, 1882)), whose fibers, upon entering the lobus protocerebrales, split into various regions of the brain to a lesser extent, and primarily extend to the tuberculum opticum (**Fig. 11** T.opt, **on Plate 19**) (anterior optic tract, (Kenyon, 1897)); and the infero-posterior fascicle (faisceau inféro-postérieur (Viallanes, 1882), posterior optic tract, (Kenyon, 1897)), most of whose fibers penetrate the protocerebrum and connect with the contralateral side as the “Cordon commissural” (Viallanes, 1887a), thus establishing a direct connection between the two lobulae.

#### Olfactory lobe

The fiber mass of the lobus olfactorius, which is covered by a layer of ganglion cells, is known to have a cortical and a neuropil layer. The cortical layer is characterized by the presence of glomerula olfactoria (**Fig. 7**, Gl.olf, **on Plate 19**), whose peripheral arrangement led (Kenyon, 1896a) to use the term “antennal morula.” These glomerula are recognized as dendritic arborizations and are particularly abundant on the upper and lower sides of the lobus olfactorius, where they form the roots for the two antennal nerves: the upper sensory (nervus antennalis superior, antenno-sensory nerve, (Kenyon, 1896a), and the lower motor (nervus antennalis inferior, antenno-motor externus, Kenyon (Kenyon, 1896a). The origin of the latter from the lower parts of the antennal morula (according to Kenyon, outside the lobus olfactorius) was confirmed by (Jonescu, 1909). Contrary to what was suggested by the latter author, my investigations show that there is no crossing of fibers from both nerves upon entering the neuropil of the lobus olfactorius.

The connections of the lobus olfactorius with other parts of the cerebrum can be grouped into two major fiber tracts: the fasciculus antennalis superior and the fasciculus antennalis inferior. The fasciculus antennalis superior mediates a connection with the ganglion cells of the pars intercerebralis and thereby with the intercerebral bridge and the ocellar nerves. It also branches off to connect with the lobus opticus of the opposite side, while the main branch extends to the lobula and medulla of the same-side lobus opticus (optic antennal branch of the fasciculus antennalis superior, (Jonescu, 1909); largely synonymous with Faisceau optico-olfactif, (Viallanes, 1882); funiculus optico-antennalis, (Haller, 1905); tractus olfactorius-opticus, (Bellonci, 1883). The fasciculus antennalis inferior, after giving off a few fibers to the lobula of the lobus opticus, ascends and, according to my investigations, also comes into contact with the central body, ultimately reaching the neuropil of the globuli. More details on the topography of these individual bundles, which are otherwise well-known, can be found in the works of the aforementioned authors.

In Tenthredinidae, the lobus olfactorius appears relatively primitive compared to the well-developed lobus opticus, both in terms of its small size (see also **Fig. 1 on Plate 18**) and the low number of glomeruli present (**Fig. 7 on Plate 19**). These insects primarily rely on their vision, while their sense of smell and touch seem to be of secondary importance.

Another characteristic feature of Tenthredinidae is the shape of the central body. Although it consists of two parts, as is the case in Hymenoptera in general, the upper part is brighter and composed of longitudinal fibers, while the lower part is darker (**Fig.7,** C.K, **on Plate 19**); however, the separation is not as distinct as in higher Apidae. Furthermore, there is not a complete encircling of the lower part by the upper cap-like portion, resulting in the central body lacking the well-known nearly semicircular shape seen in frontal sections. Instead, it appears more elongated in width and compressed dorsoventrally.

#### The ocellar nerves and intercerebral bridge

At this point, I would also like to briefly mention the course of the ocellar nerves and a peculiar commissure, the intercerebral bridge, although these conditions have been less studied in Tenthredinidae compared to Bombus and Vespa.

In general, Hymenoptera possess three ocelli (with exceptions, such as in ants), two lateral ocelli, and a median unpaired ocellus, which is considered as the fusion of two ocelli. Accordingly, two nerves originate from the median ocellus (**TextFig. 4A**, Oc.med), each of which (according to (Viallanes, 1893) is said to unite with the nerve from the adjacent lateral ocellus (Oc.lat).

**TextFig. 4A.**
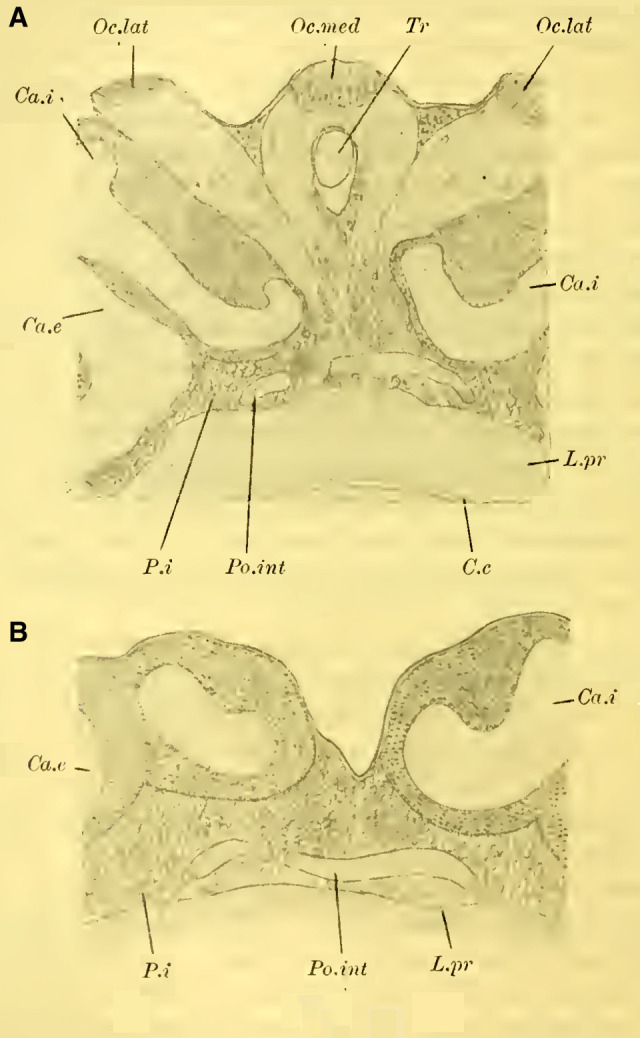
Oblique section from back-top to front-bottom through the brain of ***Bombus agrorum*** ♀. Oc.med medial ocellus, Oc.lat lateral ocelli, Tr Trachea, Ca.i inner calyx, Ca.e outer calyx, L.pr Protocerebral lobes, Po.int Pons intercerebralis, P.i Pars intercerebralis, C.c cordon commissural (Viallanes, 1887a). Scale: 1:75 **B**. Section through the brain of ***Camponotus herculeanus*** ♀. Scale: 1:145.

This representation is inaccurate. In my findings, I have discovered that the nerve fibers from the median ocellus encircle the trachea located beneath it (**TextFig. 4A**, Tr) and come to lie alongside the fibers of the lateral ocelli without uniting with them. Instead, they cross each other beneath the trachea, resulting in a complete crossing. The nerves of the lateral ocelli, initially running medially, sharply bend laterally at the edge of the mushroom body and do not participate in the aforementioned crossing. However, from what I could discern, they send a few of their medial fibers towards the midline or beyond, forming a second crossing below the first one, which is much less significant.

As (Haller, 1905) recognized, the ocellar nerve fibers then establish connections with the cells of the pars intercerebralis, whose processes further penetrate the protocerebral lobes (L.pr). Some of the fibers proceed to the nuclei of the central body (tubercules du corps central, (Viallanes, 1882); ocellar glomeruli, (Kenyon, 1897)) and establish connections with them and the central body, connecting with all the important centers of the brain. Another part of the fibers goes directly into the suboesophageal ganglion and continues into the ventral nerve cord. Additionally, I observed relationships with the “Cordon commissural,” (Viallanes, 1882) (**TextFig. 4A,** C.c), which represents the direct commissure of the fiber bundles entering the protocerebral lobes from the inner medulla of the optic lobe (Fasciculus inferior posterior) with those of the opposite side, and further connections with the fasciculus antennalis inferior, establishing a direct connection between the ocelli and both the optic lobe and the olfactory lobe.

From the processes of the cells of the pars intercerebralis, a distinct bridge-like structure is formed, which was overlooked by previous authors but was first described by (Viallanes, 1882, 1885, 1887b, 1891) not only in Hymenoptera but also in several Diptera, Orthoptera, Neuroptera, and Coleoptera as the “pont des lobes protocérébraux” (fibrillar arch, (Kenyon, 1896a)). The name chosen by (Viallanes, 1882) is justified since, indeed, in mature individuals, pathways from the protocerebral lobes penetrate the bridge, possibly indirectly involving a ganglion cell, as suggested by findings in pupae e.g.(Packard, 1881).

(Jonescu, 1909) refers to this fiber mass as the “ocellar nerve bridge” and sees it as a chiasmatic pathway of the ocellar nerves.

While the ocellar nerves do pass closely behind this bridge on their way from the ocellar ganglion to the pars intercerebralis, some fibers seem to pass through it or intersect with it, but I have never observed a direct relationship between them. Furthermore, I found this commissure to be well-developed in ***Camponotus herculeanus*** (**TextFig. 4B**, Po.int), even though this species no longer possesses ocelli or their corresponding nerve fibers. Therefore, I believe that there is no direct relationship between the bridge and the ocellar nerves. Instead, it mainly functions as a connection between a group of ganglion cells in the pars intercerebralis on one side with the corresponding group on the other side. Hence, I propose the name “intercerebral bridge” (Pons intercerebralis, Po.int).

### B. Cynipiden (gall wasps)

Examined: ***Rhodites (Diplolepis) rosae***

Due to the small size of the insects, I was unable to perform brain dissection, which is why I cannot provide a complete illustration of it. Furthermore, there are various technical difficulties in conducting the study, as the chitin in the head of freshly emerged adults is already quite hard and needs to be softened. The use of commonly used Eau de Javelle^3^ is not recommended as it can significantly damage the delicate tissue of the central nervous system. Better results were obtained using cedarwood oil, in which the heads were immersed for several days before being transferred directly to paraffin. However, even with this method, it is difficult to achieve continuous serial sections.

Judging from frontal sections (**Fig. 12 on Plate 19**), noticeable bulges are present on the dorsal surface of the brain, and accordingly, the globuli are more developed compared to the Tenthredinidae. The ganglion cells of the lobus olfactorius do not come into contact with each other in the midline, However, they have extended laterally and reach over the lobula of the optic lobe. In this case, the inner globulus is located more caudally than the outer one, which is evident from horizontal and sagittal sections (**TextFig. 5A** and **B**). As a result, the not well-defined furrow would run laterally from the back to the front in deeper sections (**TextFig. 5A**, left half) almost in the frontal plane of the brain, allowing for the distinction of an anterior lateral and a posterior medial globulus. The calyx neuropil of the internal globulus is oriented backward and somewhat medially (**TextFig. 5B**, Gl.i), while that of the external globulus is oriented forward and laterally. This behavior is characteristic of the Cynipidae and contrasts not only with the Tenthredinidae but also, as I would like to mention in advance, with all other Hymenoptera I have examined, in which the furrow runs in the opposite direction, from the back medially to the front laterally, and in a few species, directly from front to back.

**TextFig. 5A:**
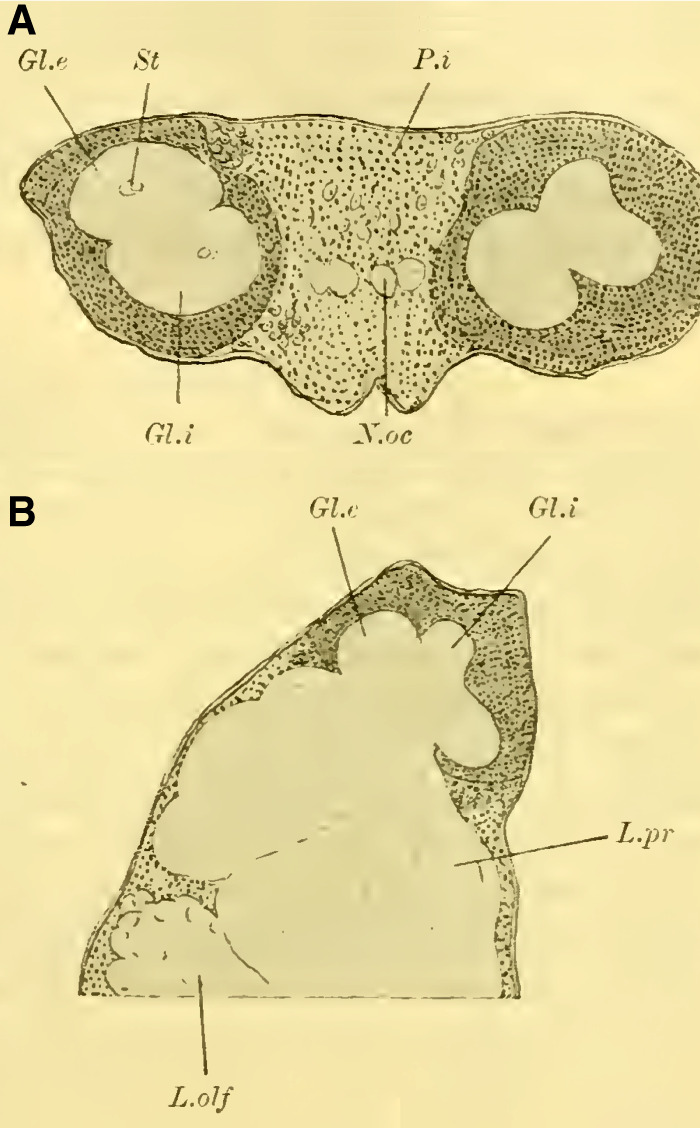
horizontal section (**A**) and sagittal cross-section (**B**) through the brain of ***Rhodites rosae*** ♀. Gl.e Outer globulus, Gl.i Inner globulus, P.i Pars intercerebralis, N.oc Ocellar nerves, L.pr Protocerebral lobes, L.olf Olfactory lobes. Scale: 1:270

The neuropile masses of the globuli have undergone differentiation, as they no longer represent simple bulbous swellings but have depressions in their center (**TextFig. 5B,** **Fig.12**). We will find this “shell type” in the Uroceridae as well. The stalks descend in a similar course as in the sawflies (compare **TextFig. 3**), and here too, at the lowest point of the described arc, the stalks cross and the anterior root (**Fig. 13** R.a, **on Plate 19**) exits directly forward, while the inner one (R.i), turning backward and slightly upward, reaches the midline below the otherwise well-developed central body (**Fig. 12,** CK, on **Plate 19**).

#### The optic lobe

seems to be significantly less voluminous relative to the protocerebrum than in the Tenthredinidae, but otherwise shows no significant differences from the conditions discussed there. However, in the horizontal section (**Fig. 13 on Plate 19**), it is apparent that the transverse diameter of the medulla (M.m.m) and lobula (M.m.i) is larger relative to the longitudinal diameter than in the sawflies, resulting in the medulla appearing more voluminous and the lobula appearing nearly circular rather than oval or elliptical. Additionally, it is worth mentioning that these two neuropiles are so close together that I was unable to determine the middle chiasma with absolute certainty, although I do not doubt its existence because of that.

#### The lobus olfactorius,

which almost covers the entire width of the protocerebrum (**Fig. 12,** L.olf, **on Plate 19**), is relatively and absolutely well-developed compared to the optic lobe and has a considerable number of glomeruli.

### C. Uroceriden

Examined: ***- Sirex gigas - Sirex invencus***

The brain of Uroceridae does not fully occupy the spacious cavity of the head, so it is relatively less voluminous compared to the smaller Tenthredinidae. However, it still appears more differentiated externally (**Fig. 2 on Plate 18**). At the protocerebrum, where the median furrow (le sillon cérébral médian) is more pronounced, there are two distinct swellings on the dorsal side, which are caused by the mushroom body. On the anterior ventral surface, the lobus olfactorius stands out due to its relative thickness, and its tip, from which the antennal nerve (N.a) emerges, is slightly directed laterally. On the lateral side, the lobus opticus (L.opt) extends somewhat downward but does not appear to be particularly well-developed compared to that of Tenthredinidae.

**The mushroom bodies**

The globuli, sitting dorsally and slightly caudally on the brain, are not completely separated from each other in Uroceridae. However, not only on horizontal sections (**TextFig. 6A**), but also on frontal sections, we can observe a distinct indentation between the two fiber masses (**Figure 14,** Sp, **on Plate 19**). However, this furrow is not clearly visible throughout its entire course. If we were to construct it on the horizontal section (**TextFig. 6A)**, it would also run diagonally from posterior-medial to anterior-lateral, with the inner globulus positioned slightly anterior to the outer one. The bilateral inner globuli do not touch in the midline, allowing the cells of the Pars intercerebralis (**Figure 14,** P.i, **on Plate 19**) located between them to expand extensively. Laterally, the globuli do not extend onto the lobus opticus, as observed in Tenthredinidae, so the large protoplasm-rich ganglion cells mentioned earlier extend from the dorsal surface of the protocerebral lobes (**Figure 14,** L.pr, **on Plate 19**) to the dorsal neurilemma of the brain.

**TextFig. 6.**
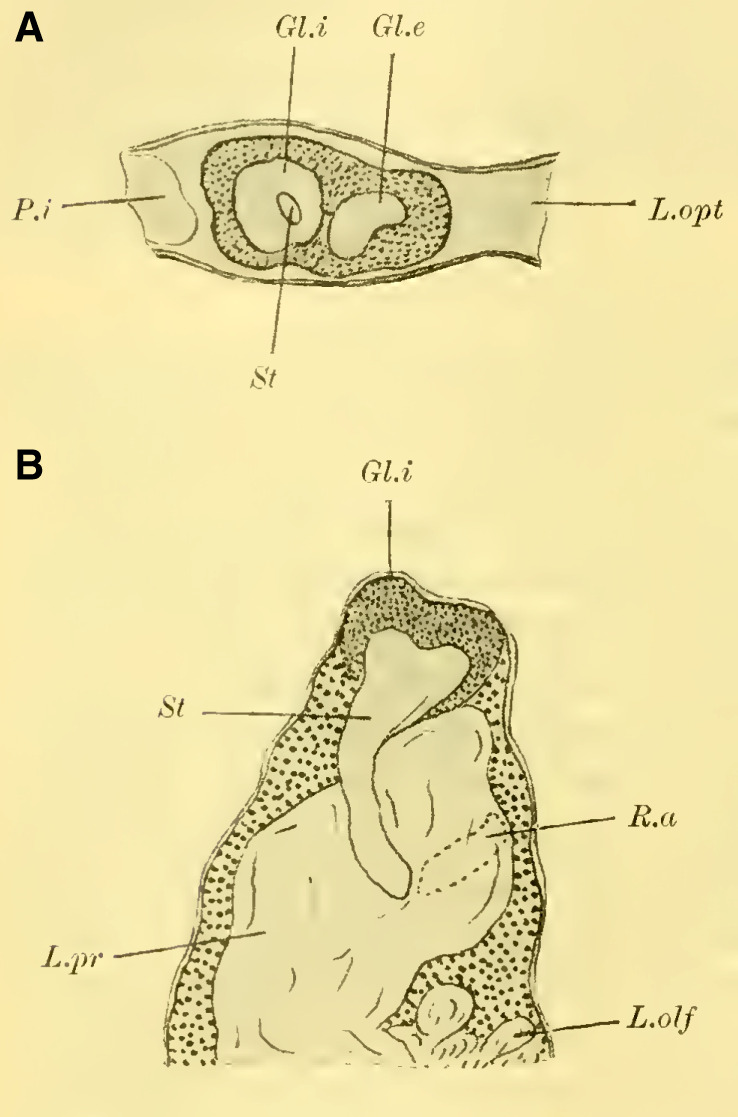
A horizontal section (**A**) and a sagittal section (**B**) of the brain of ***Sirex gigas*** ♀. Only the right half of the brain is depicted in **A**. L.opt Optic lobe, St Stem, R.a anterior root. See TextFig. 5 for other labels. Scale 1:80.

The calyx neuropil collected from the ganglion cells, similar to Cynipidae, no longer have the simple form of bulbous swellings but rather form plates that are somewhat flattened dorsoventrally, resembling circular shapes in horizontal sections (**Figure 6A**, Gl.i). They exhibit a slight depression in the middle (**TextFig. 6B,** **Figure 14 on Plate 19**), corresponding to the point of exit of the stalk, which have an elliptical to circular shape in cross-section. The longitudinally oriented fibers of the stalk are clearly demarcated from the tangentially and radially oriented fibers of the rest of the neuropil (**TextFig. 6A** St. **on Plate 18**). We can best compare this shape of the calyx to a shallow hollowed-out shell, and in this sense, we can speak of a “shell type” of the mushroom body in Uroceridae and Cynipidae, contrasting with the club-piston-shaped type found in Tenthredinidae.

Once two stalks have come together side by side, they initially descend slightly caudally, and then, after penetrating into the protocerebral lobes on the posterior upper surface (**TextFig. 6B** St), they proceed ventrally and medially towards the front, describing a curve with its concavity directed forwards and inwards. Approximately at the lowest point of this curve, as it transitions into a more horizontal course, the described crossing and separation into the inner and anterior roots occur. The anterior root ascends again (**TextFig. 6B** R.a) and bends slightly away from the midline (**Figure 15** R.a, **on Plate 19**) towards the front, ending weakly swollen beneath the cortical layer of the frontal surface of the brain. The inner root (**Figure 14** R.i, **on Plate 19**) remains at approximately the same level after the crossing but moves slightly backward again and meets its counterpart below the central body (**Figure 14** CT, **on Plate 19**).

Thus, even in Uroceridae, a complete separation of ganglion cells in the two globuli has not yet occurred, but a distinct indentation between them is already visible on frontal sections. Furthermore, we observe that in Cynipidae and Uroceridae, there is a further development in the shape of the calyx neuropil as the club-shaped type transitions into the shell type. Additionally, in Cynipidae, the globuli have extended laterally to the point where they overlap onto the protocerebral lobes and reach the lobus opticus, while the overall course of the stalks and roots is similar among all three genera discussed so far.

#### Lobus opticus

The subretinal nerve bundle layer (**Fig. 14, 15,** S. Nbs, **on Plate 19**) exhibits a very regular arrangement. The individual fiber bundles are slim and quite long, particularly those located caudally (**Fig. 15 on Plate 19**), which, due to the position of the retina, with its concave visual surface facing forward and laterally, have a greater distance to bridge to the outer lamina (M.m.e), compared to the anterior ones.

At this lamina, the same layers can be distinguished as in the Tenthredinidae. However, the lamina and its corresponding layer of cells gain significance here, especially the latter due to the large number of cells and their sharply delineated distribution. Therefore, it can be reasonably assumed that there is a fourfold stratification, which is particularly evident in frontal sections (**Fig. 14 on Plate 19**): the outer layer of ganglion cells (**Fig. 14** and **15a on Plate 19**), the spindle layer (b), the inner layer of ganglion cells (c), and the inner fiber layer (d), which can be further differentiated into the three layers mentioned in the Tenthredinidae.

Following the outer chiasma (Ch. e), which is only clearly visible in sagittal and horizontal sections and does not show anything particularly noteworthy, is the medulla. On frontal sections (**Fig. 14,** M.m.m, **on Plate 19**), it has the familiar shape of a concave-convex lens. However, on horizontal sections, it appears almost plan-convex, and its structure particularly highlights the radial striping. The lobula (M.m.i), which appears circular on frontal sections and more elliptical on horizontal sections, also exhibits clear radial striping.

When comparing the lobus opticus of the Uroceridae with that of the Tenthredinidae and Cynipidae, several notable differences emerge. The lobus opticus of the Uroceridae is both absolutely and relatively less voluminous. However, this reduction seems to have occurred not at the expense of the neuropil, but rather the fiber crossings connecting them, which is particularly evident in the case of the middle chiasma (**Fig. 14,** Ch.m, **on Plate 19**) (although this circumstance is partly due to the rotation of the neuropil that will be discussed shortly). The entire lobus opticus gives the impression of greater concentration, which may explain the separation of the lamina from the retina and the relatively long length of the subretinal nerve bundle fibers.

One condition for this concentration of optic neuropil is undoubtedly the thinness of the brain. Due to its small posterior-anterior diameter, it is no longer possible for the neuropil to stand perpendicular to the frontal plane with their largest diameter, as is still approximately the case in ***Allanthus scrophulariae*** (**Fig. 11 on Plate 19**). The lamina (**Fig. 14,** M.m.e, **on Plate 19**) mimics the shape of the retina in the frontal plane but is compressed from front to back in the horizontal plane (**Fig. 15 on Plate 19**). This results in a greater curvature compared to the sawflies, and the medially directed angle formed by the bending is smaller than a right angle, whereas in the Tenthredinidae (**Fig. 11 on Plate 19**), it is larger and approaches a flat angle.

The medulla (**Fig. 15,** M.m.m, **on Plate 19**) has undergone a rotation, so that the outer radially striped zone with its convex surface is no longer oriented laterally but almost frontally, while the flat surface faces backward and medially.

Conversely, the position of the lobula has changed. Even in the Tenthredinidae, on horizontal sections (**Fig. 11,** M.m.i, **on Plate 19**), the largest diameter of this neuropil does not align precisely perpendicular to the longitudinal axis of the brain but runs slightly from anterior lateral to posterior medial. In Sirex, this diameter has rotated to the frontal plane (**Fig. 15 on Plate 19**), causing the radially striped zone to no longer face laterally but directly backward, while the other, flatter surface seen in the sawflies is now directed frontally. As a result of these rotations, the fibers of the middle chiasma (**Fig. 15,** Ch.m, **on Plate 19**) enter the lobula from the posterior surface, while the fibers of the inner chiasma (Ch.i) mainly emerge not from the medial surface but from the inner edge of the lens. Similarly, the Fasciculus infero-anterior (not shown in **Fig. 15 on Plate 19**) exits the lobula in its lower part on the medial half of the convex anterior surface. Thus, it has shifted significantly compared to the Tenthredinidae.

#### The Lobus olfactorius

of the Uroceridae is better developed than in the Tenthredinidae, indicated not only by its larger volume but also the abundant olfactory glomeruli, which suggests a good sense of smell.

#### The Central body

(**Fig. 14,** CT, **on Plate 19**) still resembles that of the sawflies in its structure and shape: although the lower part does not completely enclose the upper part, the separation is somewhat sharper. Its shape is that of a flat-convex lens.

In both the development of the lobus olfactorius and the central body, it seems that the Cynipidae are superior to the other two forms.

### D. Ichneumonidae

Examined: ***Ichneumon obsessor - Exetastes fornicator - Aphidius rosarum***

The brain of the Ichneumonidae (**Fig. 3 on Plate 18**) appears significantly different from those of the species discussed so far, even upon external observation. The median incision on the dorsal surface of the cerebrum is well pronounced, and on both sides of it, there are bulges of brain neuropil known as the mushroom bodies, indicating a well-developed globuli. The lobus olfactorius (L.olf) also stands out due to its size, while the lobus opticus (L.opt) appears relatively less well-developed, at least in terms of its length, compared to the Tenthredinidae and Uroceridae. Through staining with borax carmine and subsequent clarification, the outline and spatial relationships of the neuropils can be distinguished even in the total preparation. Thus, at the lobus olfactorius (**Fig. 3,** L.olf, **on Plate 18**), one can observe the central neuropilar region, and at the lobus opticus, following the peripheral retina (Ret.), the lamina (M.m.e), the medulla (M.m.m), and the lobula (M.m.i), as well as their connection to the protocerebral lobes (L.pr). Additionally, at the mushroom bodies, one can see that the neuropil of the globuli takes the form of cup-like calycal structures, with the inner calyx (Calyx internus, Ca.i) positioned in front of the outer calyx (Ca.e). On both sides of the midline, the contours of the anterior roots (R.a) are visible, which, in cross-section, terminate at the anterior surface of the brain and appear approximately circular.

#### The mushroom bodies

(Flögel, 1878) states that in a species of ***Cryptus*** (Cryptinae, Ichneumomidae) the lateral calyces are positioned much further back than the inner pair; similarly, in ***Ichneumon obsessor***, one could distinguish between the anterior inner and posterior outer calyces (**TextFig. 7A and B**, Ca.i, Ca.e), with the inner calyx facing forward and medially, and the outer calyx facing backward and laterally. The walls of the same are, at least in their dorsal parts, completely separated from each other and with them the associated cell clusters, so that here for the first time a complete furrow (**Fig. 7A and B**, Sp) is present, which in this case, according to the arrangement of the calices, runs from front laterally to back medially. Additionally, the angle at which the extensions of the furrows intersect caudally from the brain gradually increases from acute to 90 degrees and beyond, particularly in the ventral regions (**Fig. 7B**). The ganglion cells of the bilateral mushroom body, which are still widely separated in Tenthredinidae, Cynipidae, and Uroceridae, come into contact with each other in the Ichneumonidae just above the ganglion cells of the Pars intercerebralis, almost in the midline.

**TextFig. 7.**
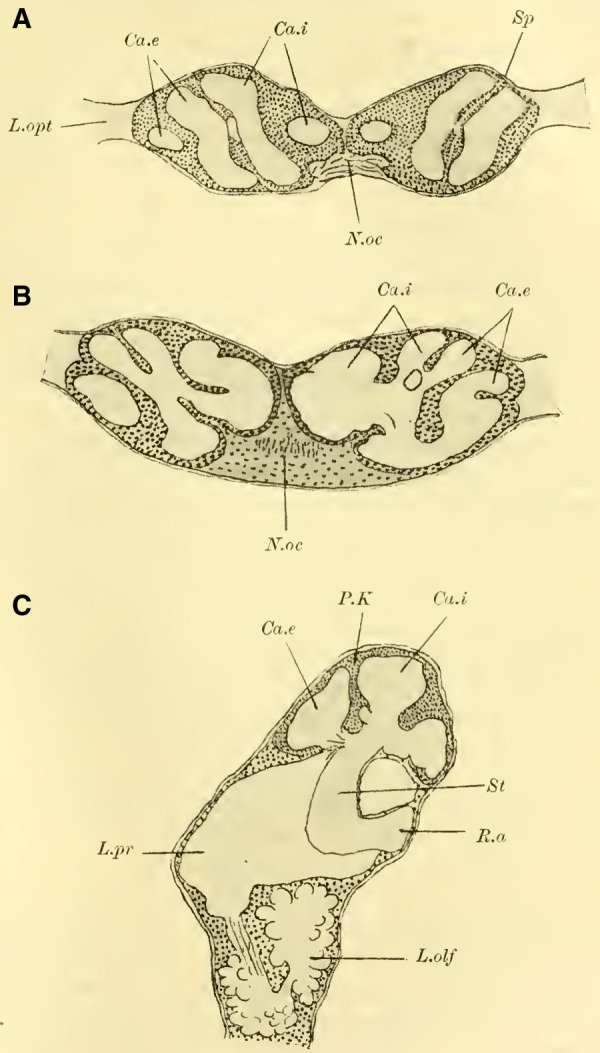
Two horizontal sections (A and B) and one sagittal section (C) of the brain of ***Ichneumon obsessor*** ♀. Ca.i Inner calyx, Ca.e Outer calyx, P.K Mushroom body, N.oc Ocellar nerves, L.opt Optic lobe, L.olf Olfactory lobe, L.pr Protocerebral lobes, R.a Anterior root, St Stem. Scale 1:75.

The walls of the neuropil of the mushroom body, which are highly developed, do not have the same thickness everywhere. There are bulges present at the upper edge (**Fig. 16 on Plate 19****, TextFig. 7C**), mostly towards the side opposite to the cavity of the calyces, giving them a shape that could be compared to a chalice. The cavities of these chalices, which are small in proportion to the massive walls, are filled with the known small ganglion cells, which also cover the outer surface up to the beginning of the stalks. As mentioned earlier, they do not come into contact with the ganglion cells of the opposite side in the midline, allowing the cells of the Pars intercerebralis to reach the dorsal surface of the brain. Laterally, the mushroom body have also expanded and overgrown the protocerebral lobes (**Fig. 16** L.pr, **on Plate 19**) until their end and the beginning of the inner neuropil of the lobus opticus (M.m.i), so that the protoplasm-rich ganglion cells, which in the previously discussed species were in a horizontal plane with those of the mushroom bodies and lobus opticus, no longer or only sporadically touch the neurilemma of the dorsal surface, but are located at the angle formed by the stalk and the protocerebral lobes.

The adjacent walls of two calyces are connected by a very strong fiber system (**Fig. 16** K, **on Plate 19**), which is also present in other Hymenoptera but appears to be particularly well-developed in Ichneumonidae.

Since the mushroom body sit directly dorsal to the protocerebral lobes, the stalks emerging from the calyces form a voluminous fiber bundle that remains of consistent size even in its further course, descending nearly in the vertical plane (**TextFig. 7C** St) and medially. The crossing takes place at the transition to a more horizontal course (**Fig. 16,** Kr, **on Plate 19**). The anterior root departs approximately perpendicularly from the stalk and goes directly forward horizontally with a short end swelling (**TextFig 7C,** **Fig. 17 on Plate 20****,** Ra), while the inner root runs horizontally slightly backward towards the midline (**Fig. 16 and 17** R.i).

#### Lobus opticus

For Ichneumonidae, the arrangement of the optic neuropils is also characteristic and differs from the patterns discussed before. The lobula, circular in frontal sections (**Fig. 16,** M.m.i, **on Plate 19**) and more oval in horizontal sections (**Fig. 17 on Plate 20****)**, is less rotated around its vertical axis compared to wood wasps; nevertheless, there is still enough rotation that the majority of the radial striping is directed caudally. The medulla (**Fig. 17**, M.m.m, **on Plate 20**) appears convex fronto-laterally and concave medio-caudally. Its shape is bean-like in frontal sections, but in horizontal sections, its diameter consistently decreases towards the medial side. The neuropil is not very voluminous; following the relatively less extended retina in the frontal plane (**Fig. 17,** Ret, **on Plate 20**), it has rotated so that its convex surface faces forward and laterally, resulting in the entire lobus opticus describing a slight anterior curve.

#### Lobus olfactorius

It is readily presumed that parasitic wasps primarily rely on their sense of smell in their interaction with the outside world, as anyone who has observed the restless, incessant movements of their antennae can attest. Indeed, I found the lobus olfactorius in Ichneumonidae to be highly developed both in terms of size and structure, surpassing any other Hymenoptera species I examined. Although the olfactory glomeruli are not very large (**Fig. 16 on Plate 19**), they are exceptionally numerous, arranged in 2-3 rows behind the periphery. The relatively voluminous

#### Central body

(**Fig. 16,** CK, **on Plate 19**), as in Cynipidae, exhibits a semicircular shape on frontal sections, with the upper dome completely overlapping the lower portion.

These characteristics, primarily studied in ***Ichneumon obsessor*** and likely applicable to all Ichneumonidae due to their similar lifestyles and instinctual development, were consistently observed in ***Exetastes fornicator*** (**TextFig. 8A**) as well. However, I cannot determine whether slight variations in size, the distance of the piliferous bodies from the midline, and other measurements exist, as investigating a more extensive sample would be required.

**TextFig. 8.**
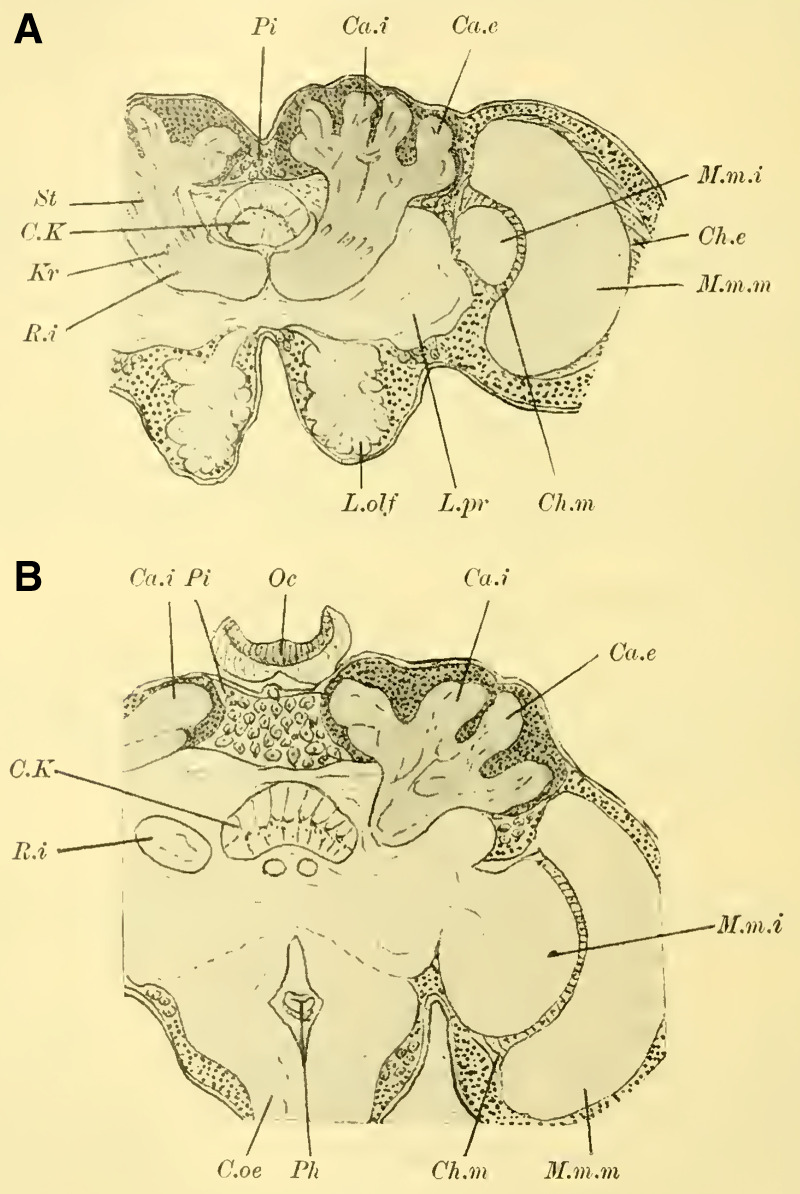
Two frontal sections of the brain of ***Exetastes fornicator* ♀** (**A**) and ***Aphidius rosarum* ♀** (**B**). P.i Pars intercerebralis, C.K Central body, R.i Inner root, Kr bifurcation of the stalks, Oc Ocellum, C.oe Subesophageal commissure, Ph Pharynx, Ch.e Outer, Ch.m Middle commissure, M.m.e lamina, M.m.m medulla, M.m.i lobula of the optic lobe. Other labels are as in the previous figures. **A**: Scale 1:52; **B**: Scale 1:140.

Such small variations appear to be present, for example, in Braconidae. (Flögel, 1878) reports that he observed the outer calyces lying almost directly behind the inner ones in a species belonging to the genus *Bracon*, although I cannot confirm this for the specie I examined, ***Aphidius rosarum*** (**TextFig. 8B**).

In this braconid wasp, the lobus opticus and lobus olfactorius exhibit the same structure as true Ichneumonidae. (The magnification of **TextFig. 8A** is 1:52, while that of **8B** is 1:140). The calyces are also constructed in the characteristic chalice-like manner and do not seem to lag behind in their relative development compared to true parasitic wasps. However, they also extend laterally over the lobus opticus but do not touch in the midline. (The depicted section is not precisely frontal but taken from top front to bottom rear, so it cannot be readily compared with the other illustrations. However, this finding is confirmed by the other sections in the series.) The mb stalks appear to be relatively less voluminous than those of the Ichneumonidae.

From the foregoing, it is evident that the brain of Ichneumonidae is significantly more differentiated than that of the previously discussed species. The furrow of the mushroom bodies is distinctly visible for the first time in its entire course, and contrary to Cynipidae, it runs in the same direction as in Tenthredinidae and Uroceridae, albeit at a much steeper angle, from posterior medial to anterior lateral. The edges of the cup-shaped neuropil of the globuli are fully developed, forming calyx-like structures with swellings on the opposite side of the calyces, extending significantly towards the midline and laterally beyond the lobus opticus. The lobus olfactorius also exhibits qualitative and quantitative development. The rotations of the neuropil (TN: the glomeruli), which are not discernible in Cynipidae but clearly visible in Uroceridae, are also present in Ichneumonidae. In addition, here is the medulla in the frontal plane corresponding to the position of the retina still shifted in front of the interior lobula.

Thus, the brain of Ichneumonidae can be considered as a further development of the type observed in wood wasps (**Uroceridae = Siricidae)** and shows more similarities suggesting closer kinship with them than with Cynipidae, although the latter appear to be more advanced than Uroceridae.

### E. Fossorial wasps and Apidae

The brains of fossorial wasps, solitary bees, and social bees are very similar to each other and are built according to a type that essentially corresponds to the one known for ***Apis mellifica*** (the honeybee). Therefore, I will first discuss the common features shared by these three groups and later address the differences and potential sexual dimorphism.

When examining the brains of fully dissected specimens (**Fig. 4, 5 on Plate 18****)**, a striking feature compared to the previously discussed groups is the robust development of the mushroom bodies on either side of the midline (P.K). Borax carmine preparations clearly reveal two distinctively shaped neuropil (Cc) on each side. Additionally, the ends of the anterior roots (R.a) can be observed as two approximately circular slight bulges on the anterior surface of the brain, just below the dorsal boundary of the protocerebral lobes (L.pr).

The optic lobe (L.opt) is well-developed, especially in higher forms (**Fig. 5 on Plate 18****, *Anthophora vulpina***). However, the olfactory lobes (L.olf) are consistently smaller than those of the Ichneumonidae, at least as large as those of the Uroceridae, and relatively larger compared to the Cynipidae and Tenthredinidae.

The paired mushroom body calyxes (globuli) in each brain hemisphere are always clearly separated from each other by the furrow (**TextFig. 9** Sp), which does not run from lateral to medial (as in Cynipidae), but in most cases, similar to other Hymenoptera, it runs from medial to lateral, often at an oblique angle (***Xylocopa violacea***, **TextFig. 9B**) or less oblique angles (***Crabro cribrarius***, ***Anthophora vulpina***, **TextFig. 9A and C**). In rare cases, as will be discussed later, the furrow almost runs straight from posterior to anterior, allowing for a distinction between two medial (possibly anterior) and two lateral (possibly posterior) lobes.

**TextFig. 9.**
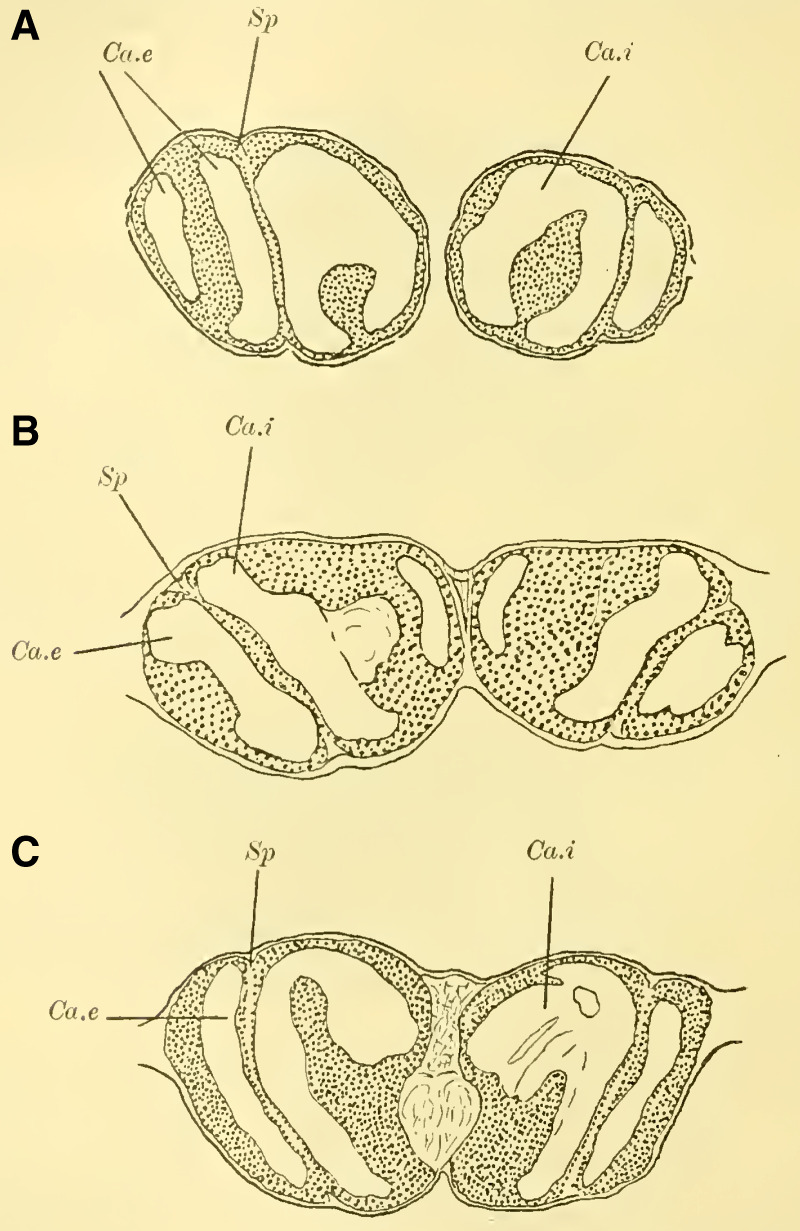
Three horizontal sections of the brain of ***Crabro cribrarius*** ♀ (**A**), ***Xylocopa violacea*** ♀ (**B**), and *Anthophora vulpina* ♀ (C). Ca.e outer calyx, Ca.i inner calyx, Sp furrow. Scale A 1:60; B and C 1:40

The ganglion cells within each lobe shows further differentiation. Frontal sections (**Fig. 19,** and subsequent **on Plate 20**) reveal two lateral groups of cells, which appear less stainable compared to the medial group. However, in reality, there is only one group that forms a ring around the medial portion (Haller, 1905). I believe these middle groups to be identical to the “formation centers” of ganglion cells described by (Bauer, 1904) in the inner metamorphosis of the central nervous system of insects. From these centers, the other cells of the mushroom bodies originate during post-embryonic development. According to (Jonescu, 1909), the processes of the middle group form the medial parts of the stalks, while the processes of the lateral group form the peripheral parts. The fibrillar neuropil of the lobes is derived from the processes of the cells that cover it on the outer surface in adult individuals.

The neuropil of the mushroom bodies assumes the characteristic shape of a cup, or calyx, after (Newton, 1879)^4^, and its walls can exhibit swellings and thickenings, particularly at the upper edge, thereby increasing the surface area. Corresponding to the arrangement of the associated ganglion cells and the course of the furrow, two outer (posterior) calyces (**Fig. 19ff.** Ca. e, **on Plate 20**) and two inner (anterior) calyces (Ca. i) can be distinguished.

From the base of each calyx, the stalks (St) descend together until they reach the level of the central body (CK), where they cross (**Fig. 19,** Kr, **on Plate 20**). From this point, the anterior root (**Fig. 18,** R.a, **on Plate 20**) projects forward with a slight bulge, while the inner root (**Fig. 19ff.** R.i, **on Plate 20**) extends medially until it meets the corresponding root from the opposite side beneath the central body.

#### The optic lobe

is structured in a typical manner. The medulla (**Fig. 18,** M.m.m, **on Plate 20**) and lobula (M.m.i) exhibit rotations similar to those observed in Uroceridae and Ichneumonidae, albeit to varying degrees. The medulla faces more anteriorly with its concavity, while the lobula faces more posteriorly. The structure of the

#### Olfactory lobe

is the same as in Tenthredinidae. Differences among genera were found only in terms of the size and number of olfactory glomeruli.

When comparing brains that are built in essentially the same way, it was expected that differences would primarily manifest in the size ratios of different parts of the brain rather than the overall size. However, estimating these ratios is not always easy when examining whole series of sections, and it becomes even more challenging to illustrate them with figures. Comparing individual selected sections, which are not uniformly oriented due to the limited availability of material, can lead to erroneous conclusions. Therefore, in order to obtain as objectively comparable results as possible, I conducted various measurements on the brains. (Dujardin, 1850) previously performed similar measurements to a limited extent by considering the brain as a rotational ellipsoid, excluding a portion of the optic lobes, and calculating its volume based on the three main axes. He then compared this volume to the volume of the entire insect body determined by water displacement. However, in my opinion, it is more important to determine the size ratio of specific parts of the cerebrum, particularly the mushroom bodies as the main reflex centers, in relation to the other parts. I did not pursue volume measurements due to technical reasons, as I believe that usable values can be obtained using linear measurements. For these measurements, the following parameters were used (see schematic in **TextFig. 10**):

1. The maximum width of the mushroom bodies = a, indicated on the diagram by the dotted line a-a. If the mushroom bodies did not touch at the midline, this measurement was determined as 2 x a/2.
2. The maximum width of the protocerebral lobes: b (schematic b-b).
3. The maximum distance between the medullas of the optic lobes: c (schematic c-c).
4. The maximum height of the mushroom bodies = h (schematic h).

The measurements a and h (greatest width and height of the mushroom bodies) provided a useful basis for comparing the configuration of the mushroom bodies.

**TextFig. 10.**
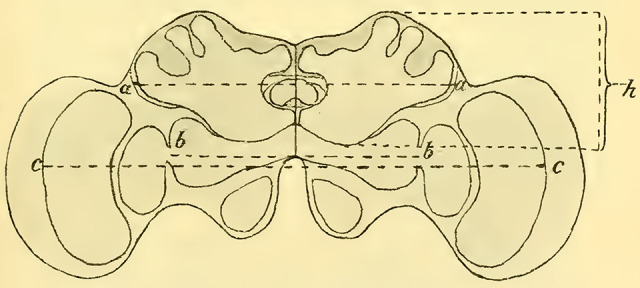
Diagram for the various measurements: a -- a = a: greatest width of the mushroom bodies, b -- b = b: greatest width of the protocerebral lobes, c -- c = c: greatest distance between the medullae of the lobus opticus, h: greatest height of the mushroom bodies.

It was more challenging to determine linear measurements that could provide an approximate idea of the size of the rest of the brain and allow for a comparison with the extent of the mushroom bodies. The maximum width of the protocerebral lobes (b) alone would not be sufficient, as in some males, the neuropil of the optic lobes become relatively substantial and can significantly influence the ratio of the size of the mushroom bodies to that of the rest of the cerebrum. Therefore, I also considered the maximum distance between the medullae of the lobus opticus (c) for comparison. The height measurement is only applicable to approximately similarly oriented sections, but measurements 1-3 are independent of that and can usually be determined with reasonable accuracy. However, it is advisable to take repeated measurements and use the arithmetic mean. From these four absolute measurements, the following four ratios can be derived: („Indices”):

1. a/b: Ratio of the width of the mushroom bodies to the width of the protocerebral lobes.
2. a/c: Ratio of the width of the mushroom bodies to the distance between the medullae of the lobus opticus.
3. h/b: Ratio of the height of the mushroom bodies to the width of the protocerebral lobes.
4. h/c: Ratio of the height of the mushroom bodies to the distance between the medullae of the lobus opticus.

These indices will increase as the mushroom bodies grow larger in relation to the protocerebral lobes and the medullae of the lobus opticus. Additionally, as the height of the mushroom bodies increases relative to the other measurements, the indices will also increase.

### a) Fossorial wasps and Archiapidae

I have only examined ***Crabro cribrarius*** (**TextFig. 11A)** and ***Cerceris labiata*** among the fossorial species. The depicted section is slightly angled from top-rear to bottom-front, making a direct comparison with **TextFigs. 11B and C** more difficult. However, it is sufficient to show that the brain is built according to the *Apis* type. The four calyces (Ca) are well-developed and extend laterally over the lobus opticus without reaching the medulla (M.m.m.), as seen in ***Prosopis*** (**TextFig. 11B**). However, the two inner globuli do touch in the midline, although not completely in ***Prosopis*** (***Hylaeus,* Colletidae)** and ***Sphecodes*** (**cuckoo bee, Halictidae**).

**TextFig. 11.**
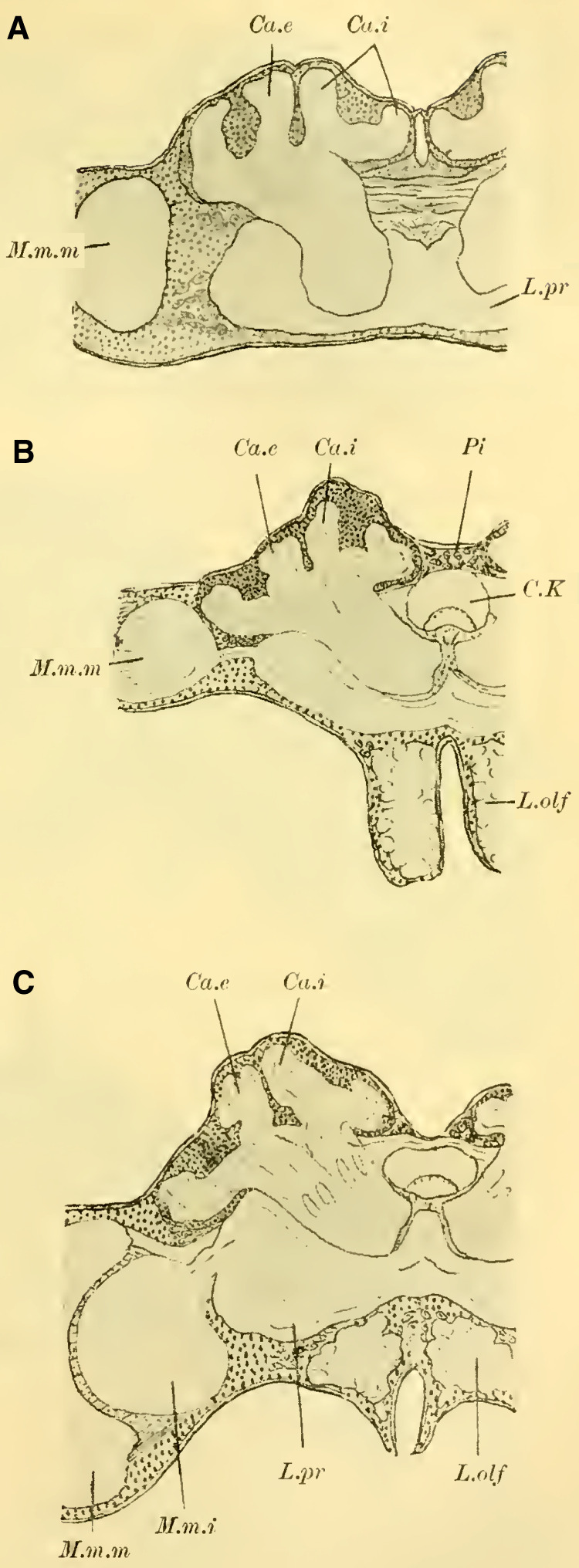
Three frontal sections through the brain of ***Crabro cribrarius*** (**A**), ***Prosopis variegata*** ♀ (**B**), and ***Sphecodes gibbus*** ♀ (**C**). Ca.e: outer calyx, Ca.i: inner calyx, M.m.i: lobula, M.m.m: medulla, L.pr: protocerebral lobes, L.olf: olfactory lobes, C.K: central body, P: pars intercerebralis. Magnification: **A** and **C**: 1:60; **B**: 1:75.

The last two species appear to be roughly similar in terms of the development of their central nervous system, as indicated in **Table I.** While the brain of ***Sphecodes*** is larger in absolute terms (except for c), the mushroom bodies of Prosopis are relatively well-developed, resulting in two indices (a/b, h/b) being higher in Prosopis compared to the other two indices.

**Table I:** 

	a (µ)	b (µ)	c (µ)	h (µ)	a/b	a/c	h/b	h/c
Cabro cribarirus ♀	911,4	824,6	1649,2	390,6	1,10	0,552	0,473	0,256
Prosopis variegata ♀	694,4	607,6	2215,2	303,8	1,14	0,313	0,500	0,137
Shecodes gibbus ♀	889,7	868,0	1822,8	379,75	1,02	0,488	0,437	0,208

a = greatest width of mushroom bodies line a-a

b = greatest width of the protocerebral lobes line b-b

c = greatest distance between the medullae of the optic lobes line c-c

h = greatest height of the mushroom bodies line h

These four measurements are given in micrometers (µm). From these measurements, the ratio values or “indices” indicated as a/b, a/c, h/b, h/c are derived”.

Unfortunately, I did not have access to males of these species.

### b) Gastriligidae und Podiligidae

In the further discussion, I would like to focus on the group of species that (Friese, 1891) derives from Prosopis in his phylogenetic table, including Ceratina, Colletes, Xylocopa, and the entire group of abdomen-collecting bees (Gastriligidae). Regarding the accompanying **Table II**, it should be noted that the numbers indicated under D a/b, D a/c, etc., represent the difference in the respective indices between females and males. A positive sign indicates an advantage for females, while a negative sign indicates a disadvantage compared to males.

**Table II:** 

	a (µ)	b (µ)	c (µ)	h (µ)	a/b	a/c	h/b	h/c	D a/b	D a/c	D h/b	D h/c
Ceratina cucurbitina♀	781,2	726,95	1215,2	347,2	1,07	0,642	0,477	0,256	.	.	.	.
Colletes cunnicularius ♀	1475,6	1302,0	2690,8	477,4	1,13	0,511	0,366	0,177	}+0,01	+0,002	−0,014	+0,005
„ „ ♂	1215,2	1085,0	2387,0	412,3	1,12	0,509	0,380	0,172				
„ davicranus ♀	1128,4	954,8	1996,4	390,6	1,20	0,576	0,409	0,195	}−0,002	0,002	0,062	0,031
„ „ ♂	1171,8	954,8	2039,8	336,35	1,22	0,574	0,347	0,164				
Xylocopa violacea ♀	1900,0	1625,0	3850,0	750,0	1,16	0,493	0,461	0,194	}+0,04	−0,028	0,037	−0,003
„ „ ♂	1605,8	1432,2	3081,4	607,6	1,12	0,521	0,424	0,197				
Eriades crenulatus ♀	998,2	770,35	1345,4	347,2	1,27	0,741	0,444	0,258	}+0,02	.	−0,009	.
„ „ ♂	868,0	694,4	.	314,65	1.25	.	0,453	.				
Osmia cornuta ♀	1453,9	1204,35	2734,2	564,2	1,20	0,531	0,468	0,206	}0,00	+0,040	0,000	0,016
„ „ ♂	1258,6	1041,6	2560,6	488,25	1,20	0,491	0,468	0,190				
Chalicodoma muraria ♀	1627,5	1367,1	2821,0	651,0	1,20	0,584	0,476	0,230	}+0,10	.	0,039	.
„ „ ♂	1150,1	1041,6	.	455,7	1,10	.	0,437	.				
Megachile lagopoda ♀	1432,2	1182,6	2387,0	629,3	1,21	0,600	0,532	0,263	}+0,06	+0,124	+0,023	+0,053
„ „ ♂	1302,0	1128,4	2734,2	575,05	1,15	0,476	0,509	0,210				
Anthidium 7-dentatum ♀	1497,3	1204,35	2473,8	651,0	1,24	0,605	0,540	0,263	}+0,12	+0,051	+0,040	+0,016
„ „ ♂	1215,2	1085,0	2191,7	542,5	1,12	0,554	0,500	0,247				

The last four headings, labelled D a/b, D a/c, D h/b, D h/c, indicate the differences that exist between the indices of ♀ and ♂, namely that the positive sign means that the female has the advantage, while the negative sign indicates that it is at a disadvantage compared to the male. The other designations are as in Table 1.

Upon comparing the figures, certain differences are already noticeable. The mushroom body are still low in ***Colletes*** (**Fig. 19 on Plate 20****; TextFig. 12**), and the stalks are less voluminous. In contrast, there is already some progress towards development in ***Ceratina cucurbitina*** (**TextFig. 13 A**) and ***Eriades crenulatus*** (**TextFig. 13 B**). ***Xylocopa violacea*** (**Fig. 20 on Plate 20**) stands out particularly due to the significant development of the Lobi optici, allowing the well-developed cups (calyces) to extend only up to the medial part of the lobula (M.m.i).

**TextFig. 12.**
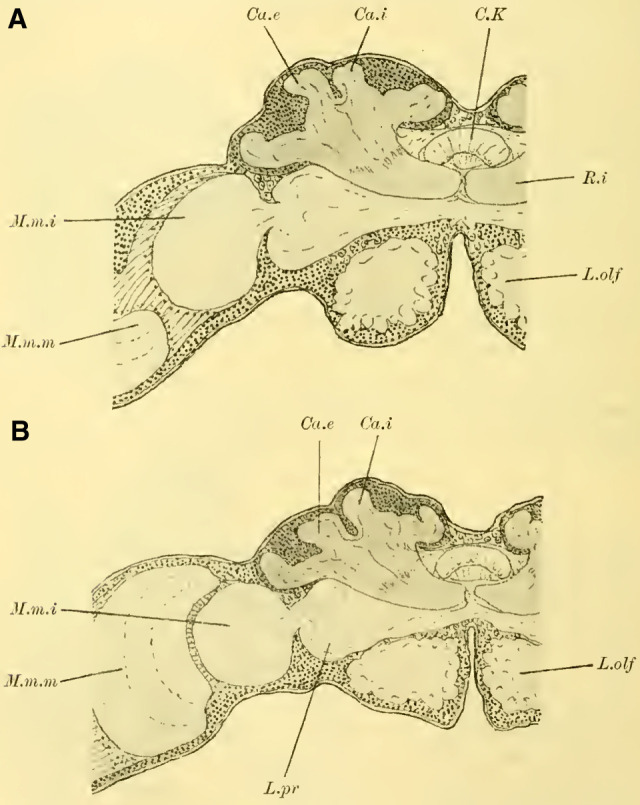
Two frontal sections through the brain of ***Colletes cunnicularius*** ♀ (**A**) and ♂ (**B**). Ca.e outer calyx, Ca.i inner calyx, M.m.m medulla, M.m.i lobula, C.K central body, L.olf olfactory lobe, L.pr protocerebral lobe. Scale: 1:52.

**TextFig. 13.**
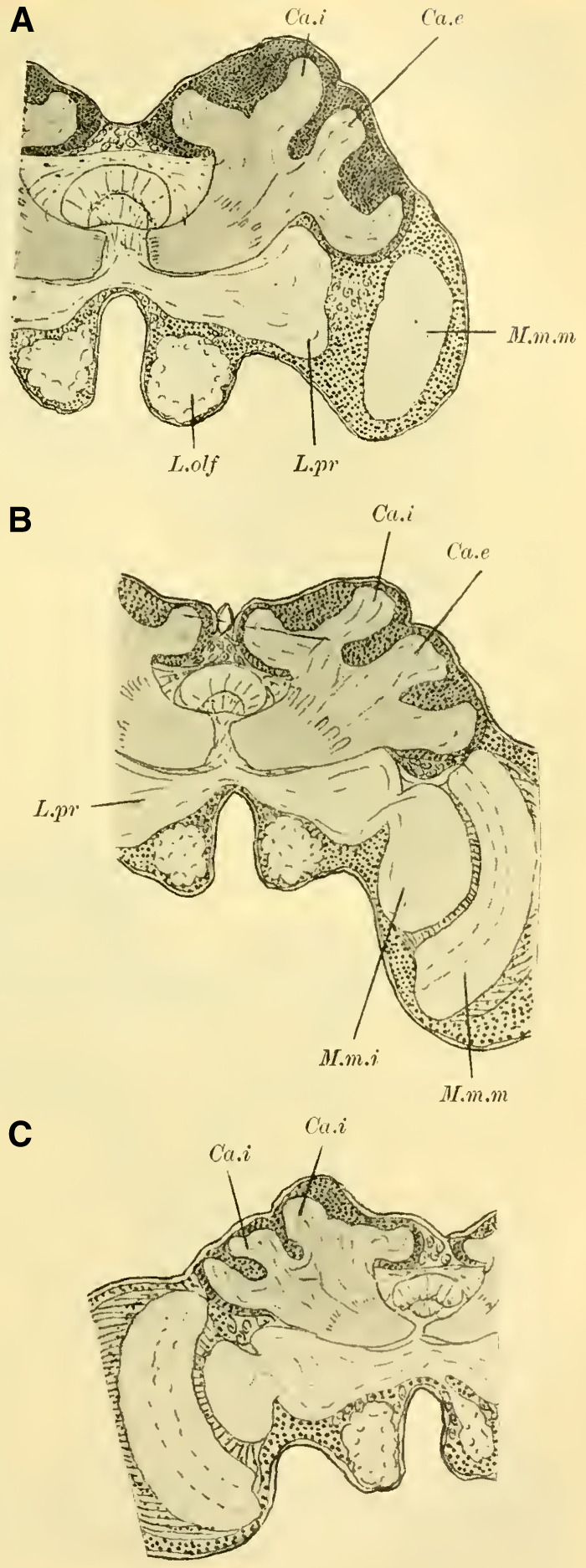
Three frontal sections through the brain of ***Ceratina cucurbitina*** ♀ (**A**) ***Eriades crenulatus*** ♀ (**B**) and ♂ (**C**). Labels as in TextFig. **12**. C = slightly schematic. A = 1:80. B and C = 1:60.

Among the following species: ***Osmia cornuta*** (**Fig. 21 on Plate 20**), ***Chalicodoma muraria*** (**Fig. 22 on Plate 20**), and ***Älegachile lagopoda*** (**Fig. 23 on Plate 20**), the tendency of the mushroom body to grow upward and laterally becomes more pronounced. In the most highly developed species of this group, ***Anthidium septemdentatum*** (**Fig. 24 on Plate 20**), the lateral edge of the globuli reaches the medulla, M.m.m). The Lobus opticus also increases in size, although, with the exception of *Xyclocopa*, not in the same proportion as the mushroom body, as indicated by the numbers in **Table II**. From these it follows that the indices can vary between two species of the same genus, such as ***Colletes cunnicularius*** ♀ and ***Colletes davicranus*** ♀, and that a completely consistent series cannot be established among the lower forms. However, in ***Osmia cornuta*** ♀, all four indices for females increase in a nearly uniform sequence.

In addition to these differences among genera and species, sexual dimorphisms are also present in all species for which males were available. They may not be readily apparent in lower species, apart from absolute size differences (**TextFig. 13B and C**), but the measurements indicate that in Colletes cunnicularius (**TextFig. 12**), females surpass males in terms of the relative width of the mushroom body, while males have greater height. In contrast, in Colletes davicranus (**Table II**), males, whose brains are larger than those of females, surpass them in the width of the mushroom body but fall behind in terms of height. The situation is different in Xylocopa violacea (**TextFig. 14A, B**), where the Lobus opticus of the female is more developed than that of the male, causing the mushroom body of the female to be relatively smaller compared to the overall size of the brain (a/c, h/c), but larger in relation to the protocerebral lobes (a/b, h/b).

**TextFig. 14.**
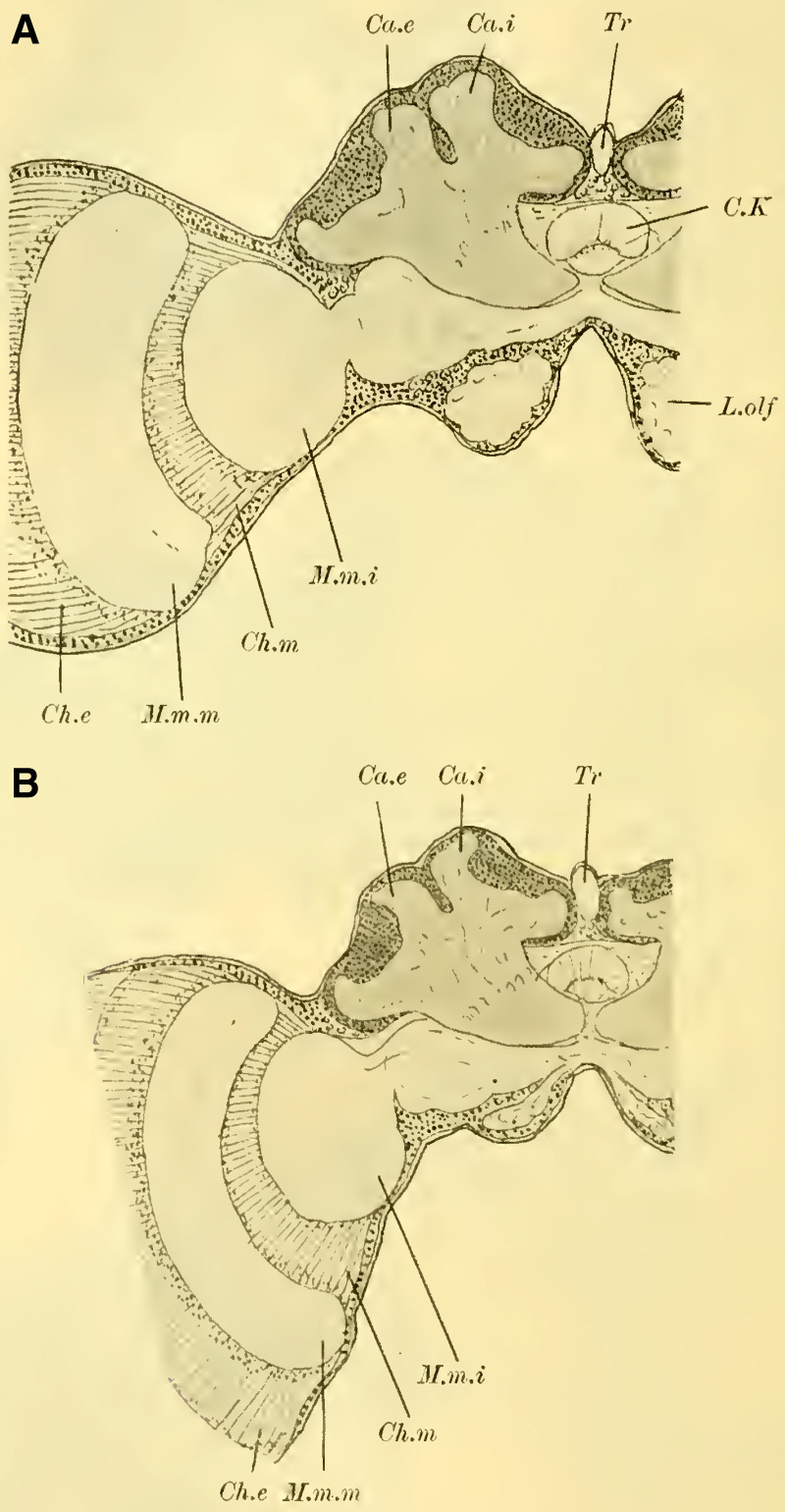
Two frontal sections through the brain of ***Xylocopa violacea*** ♀ (**A**) and ♂ (**B**). Tr Trachea, Ch.e outer cross, Ch.m middle cross. The other labels as in TextFig. 12. Scale: 1:32.

For ***Eriades crenulatus*** (**TextFig. 13B, C**), I was unable to fully conduct the comparison as the male's Lobus opticus was mutilated during preparation, but it seems that the male surpasses the female only in the h/b ratio.

Starting from Osmia cornuta (**TextFig. 15A and B**), it is now evident that the female is, in contrast to the male, superior in all four indices, or at the very least, equal. This distinction is particularly notable, as, unlike Xylocopa, the lobus opticus is considerably larger in the male of Osmia cornuta than in the female.

**TextFig. 15.**
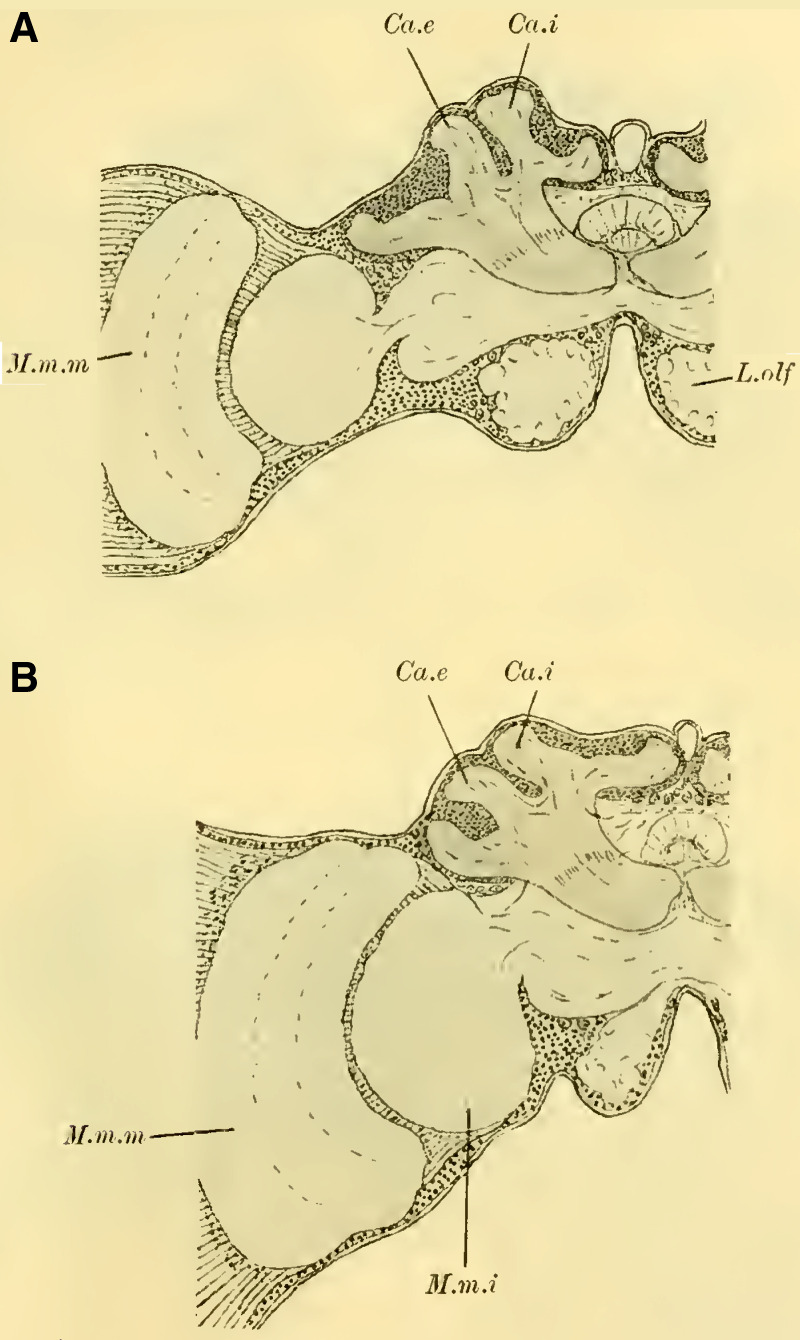
Two frontal sections through the brain of ***Osmia cornuta*** ♀ (**A**) and ♂ (**B**). Labels as in TextFig. 12. Scale: 1:40.

The more significant development of the lobus opticus in males, resulting in narrower and shorter mushroom bodies, which is likely present in ***Chalicodoma muraria*** as well but not reliably detectable for the same reason as in ***Eriades*** (Heriades, Megachilidae) becomes even more pronounced in ***Megachile lagopoda*** (**TextFig. 16A, B**), where the female is superior to the male, particularly in terms of the width of the mushroom body. This is also evident in ***Anthidium septemdentatum*** (**TextFig. 17A, B**), which represents the most highly developed form in this series. In this genus, it was also observed that sexual dimorphism is not only evident in the size of the globuli and calyces but also in their arrangement, as in the male the furrow is significantly more inclined compared to the female. (***Anthidium oblongatum***, **TextFig. 18).**

**TextFig. 16.**
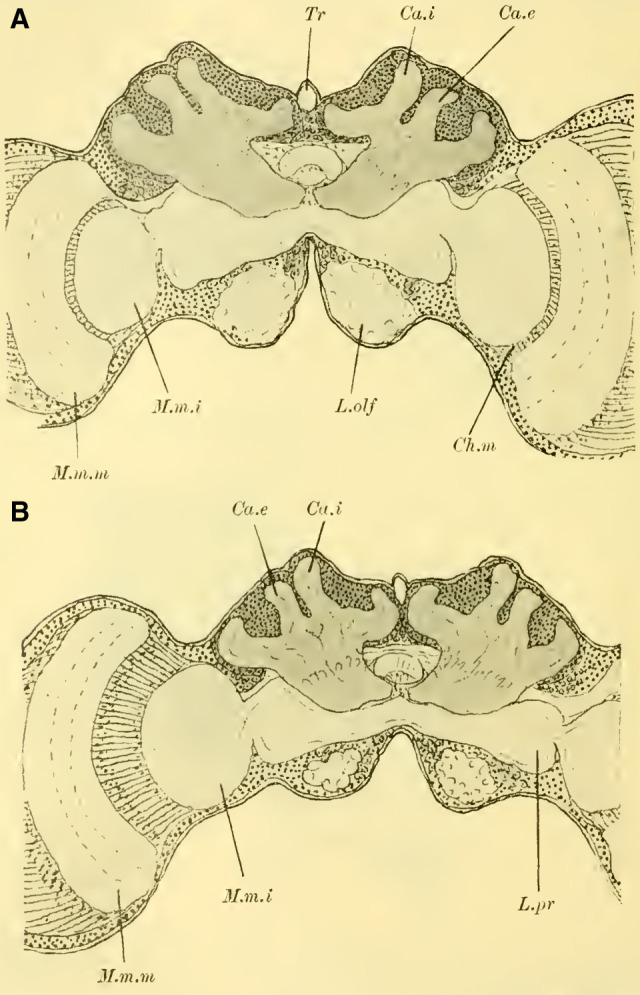
Two frontal sections through the brain of ***Megachile lagopoda*** ♀ (**A**) and ♂ (**B**). Labels as in TextFig. 12. Scale: 1:40.

**TextFig. 17.**
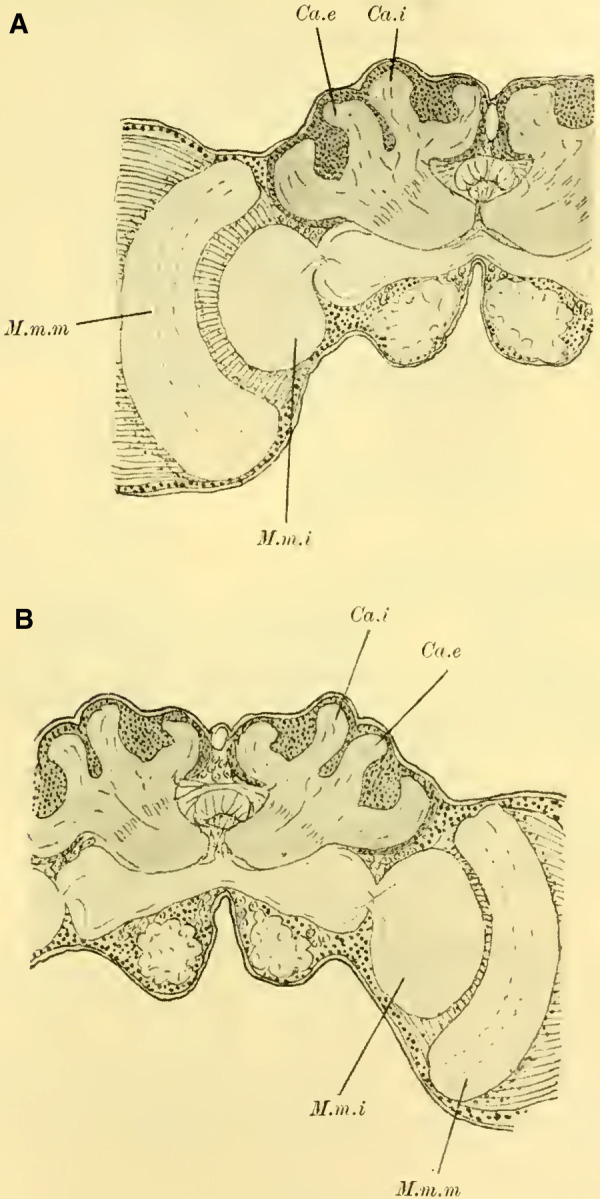
Two frontal sections through the brain of ***Anthidium septemdentatum*** ♀ (A) and ♂ (B). Labels as in TextFig. 12. Scale: 1:40.

**TextFig. 18.**
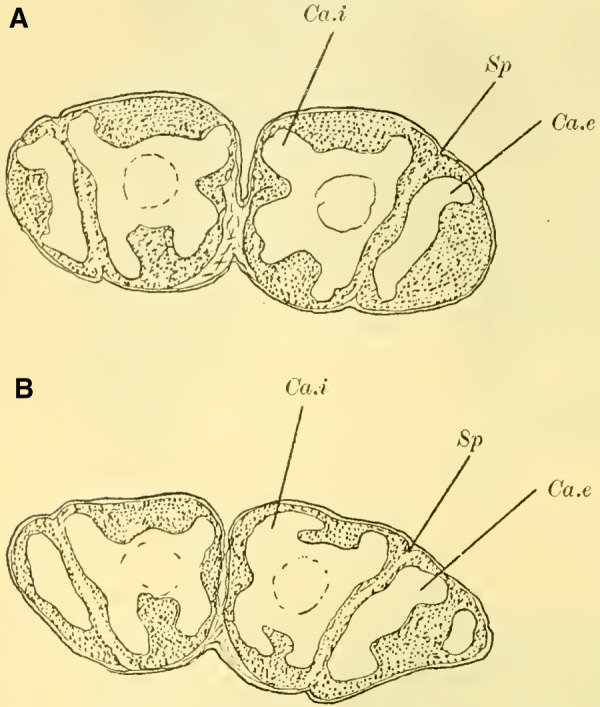
Two horizontal sections through the brain of ***Anthidium oblongatum*** ♀ (**A**) and ♂ (**B**). Sp furrow. Scale: 1:45.

I had limited material available for representatives of the second group, which (Friese, 1891) derives from ***Sphecodes*** and includes the majority of Podilegidae (TN: carrying pollen on their legs). However, I believe that the material was sufficient to establish the fundamental relationships present in this group.

Here too, differences are already present in lower species, as can be observed in the comparison of sections through the brains of ***Andrena fulva*** (**Fig. 25 on Plate 20**) and ***Halictus quadristrigatus*** (**Fig. 26 on Plate 20**). In the former, the mushroom body have a considerable height but extend laterally only up to about the middle of the lobula (M.m.i) of the well-developed optic lobe, while in ***Halictus***, they are less tall and more extended in width, which is also reflected in the measurements provided in **Table III**.

**Table III:** 

	a (µ)	b (µ)	c (µ)	h (µ)	a/b	a/c	h/b	h/b	D a/b	D a/c	D h/b	D h/c
Halictus 4-strigatus ♀	1106,7	954,8	1822,8	466,55	1,15	0,607	0,488	0,255	-	-	-	-
„ calceatus ♀	824,6	694,4	1323,7	325,5	1,18	0,622	0,468	0,245	}−0,02	-	+0,012	-
„ „ ♂	1019,9	856,6	-	390,6	1,20	-	0,456	-				
Andrena fulva ♀	1388,8	1171,8	2538,9	531,65	1,18	0,547	0,453	0,209	-	-	-	-
„ albicans ♀	1041,6	954,8	2039,8	477,4	1,09	0,510	0,500	0,234	}−0,02	+0,034	0,000	+0,020
„ „ ♂	868,0	781,2	1822,8	390,6	1,11	0,476	0,500	0,214				
„ carbonaria ♀	1193,5	1106,7	2517,2	477,4	1,07	0,474	0,431	0,189	}−0,02	+0,009	+0,059	+0,032
„ „ ♂	1085,0	989,52	2343,6	368,9	1,09	0,465	0,372	0,157				
Nomia diversipes ♀	976,5	868,0	-	434,0	1,12	-	0,500	-	}+0,01	-	+0,067	-
„ „ ♂	1085,0	976,5	-	423,15	1,11	-	0,433	-				
Systropha curvicornis ♀	998,2	824,6	-	336,35	1,21	-	0,407	-	-	-	-	-
Eucera longicornis ♀	1345,4	1085,0	2387,0	542,5	1,24	0,563	0,500	0,227	}+0,08	0,000	+0,020	−0,006
„ „ ♂	1258,6	1085,0	2235,1	520,8	1,16	0,563	0,480	0,233				
Anthophora vulpina ♀	1562,4	1171,8	2473,8	672,7	1,33	0,631	0,574	0,271	-	-	-	-

Labels as in Table I and II

On the other hand, ***Nomia diversipes*** (**TextFig. 19A**) demonstrates a superior relative height of the mushroom body compared to the aforementioned species, whereas in ***Systropha curvicornis*** (**TextFig. 19B**), the development has primarily occurred laterally. (By the way, the last object was poorly oriented, and I believe that the height is greater than what I could measure under these circumstances).

**TextFig. 19.**
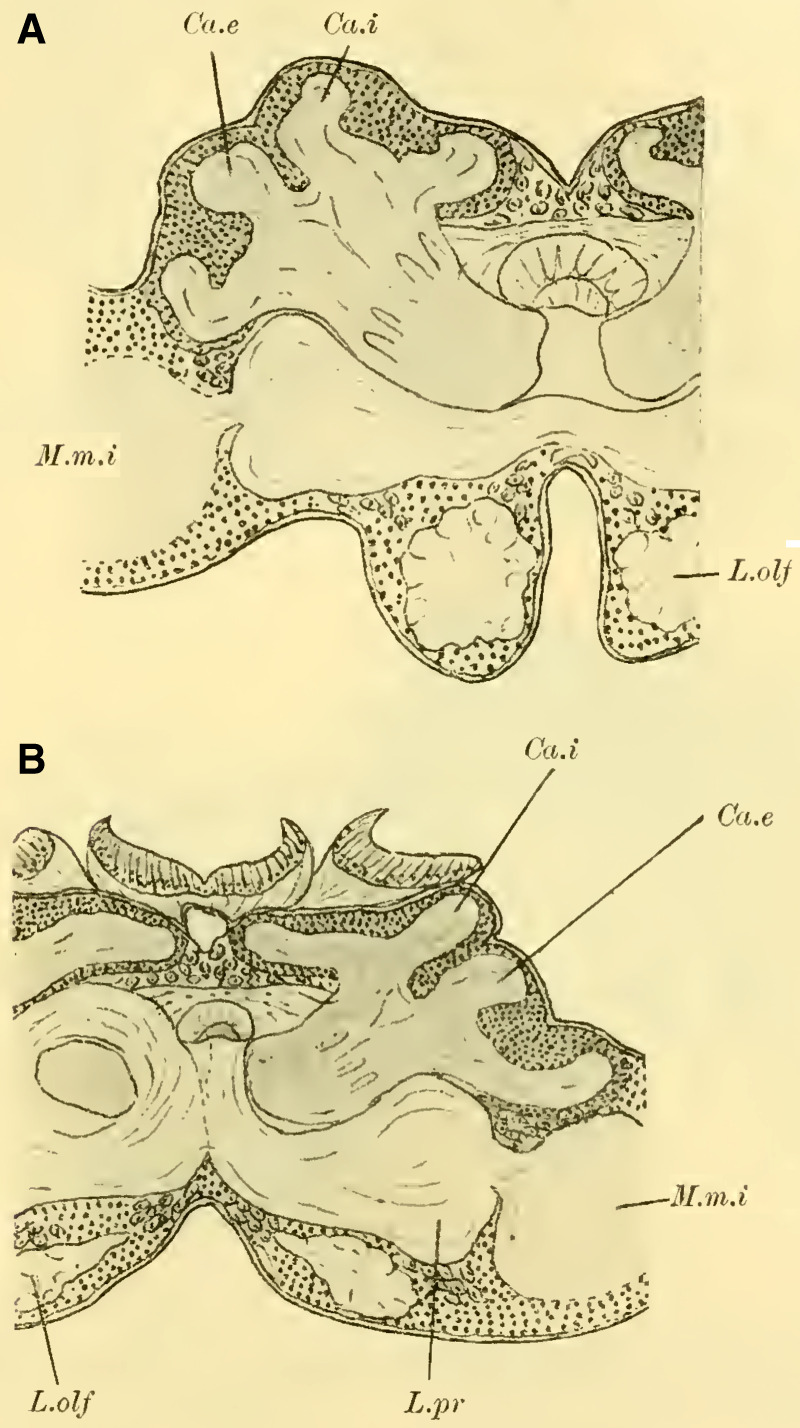
Two frontal sections through the brain of ***Nomia diversipes*** ♀ **(A**) and ***Systropha curvicornis*** ♀ (B). The labels are the same as in TextFig. 12. Scale: 1:75.

Even among species of the same genus, differences can be observed, without one species being considered more developed than the other. For example, in ***Andrena albicans*** ♀, there is a relatively narrower width of the mushroom body compared to ***Andrena fulva*** ♀, but this is compensated by a greater height.

***Eucera longicornis*** (**Fig. 27 on Plate 21**), on the other hand, surpasses the previous species in almost all indices and is further surpassed by ***Anthophora vulpina*** (**Fig. 28 on Plate 21**), which likely represents the most highly developed genus among solitary apid bees. Its indices consistently exceed those of ***Anthidium*** (**Table II**) and approach those of bumblebees (**Table IV**), with which ***Anthophora's*** brain shares a great similarity.

**Table IV:** 

	a (µ)	b (µ)	c (µ)	h (µ)	a/b	a/c	h/b	h/b
Bombus lapidarius ♀	1692,6	1215,2	2300,2	629,3	1,38	0,736	0,517	0,273
„ „ ☿	1432,2	1128,4	1887,9	499,1	1,26	0,755	0,442	0,264
„ „ ♂	1345,4	998,2	2039,8	629,3	1,34	0,659	0,565	0,276
„ agrorum ♀	1605,8	1128,4	2083,2	694,4	1,42	0,770	0,615	0,333
„ „ ☿	1649,2	1171,8	1996,4	585,9	1,40	0,826	0,500	0,293
„ terrestris ☿	1822,8	1258,6	2560,6	846,3	1,44	0,711	0,672	0,330
„ „ ♂	1562,4	1171,8	2560,6	759,5	1,33	0,610	0,648	0,296
Apis mellifica ♀	1171,8	1041,6	1866,2	520,8	1,12	0,627	0,500	0,279
„ „ ☿	1215,2	1085,0	2039,8	607,6	1,12	0,595	0,560	0,297
„ „ ♂	1128,4	1041,6	2126,6	585,9	1,08	0,536	0,562	0,275

Labels as in Table I and II

Sexual dimorphism can already be found in ***Halictus*** and ***Andrena*** (**TextFig. 20**). In ***Andrena***, the male is approximately at the same level as the female due to the smaller relative width of the protocerebral lobes. However, in ***Andrena carbonaria*** (see **Table III**), the female has an advantage. This is likely the case in ***Nomia diversipes*** and ***Eucera longicornis*** as well (**TextFig. 21**). However, the situation here is not as clear as in the case of the Gastrilegidae, as even in the highly developed ***Eucera***, where the female has well-developed optic lobes, the male is superior to the female in terms of the ratio of the mushroom body to the greatest distance between the medullae. I cannot provide information about the conditions in *Anthophora* because I did not have enough males and females of the same species available.

**TextFig. 20.**
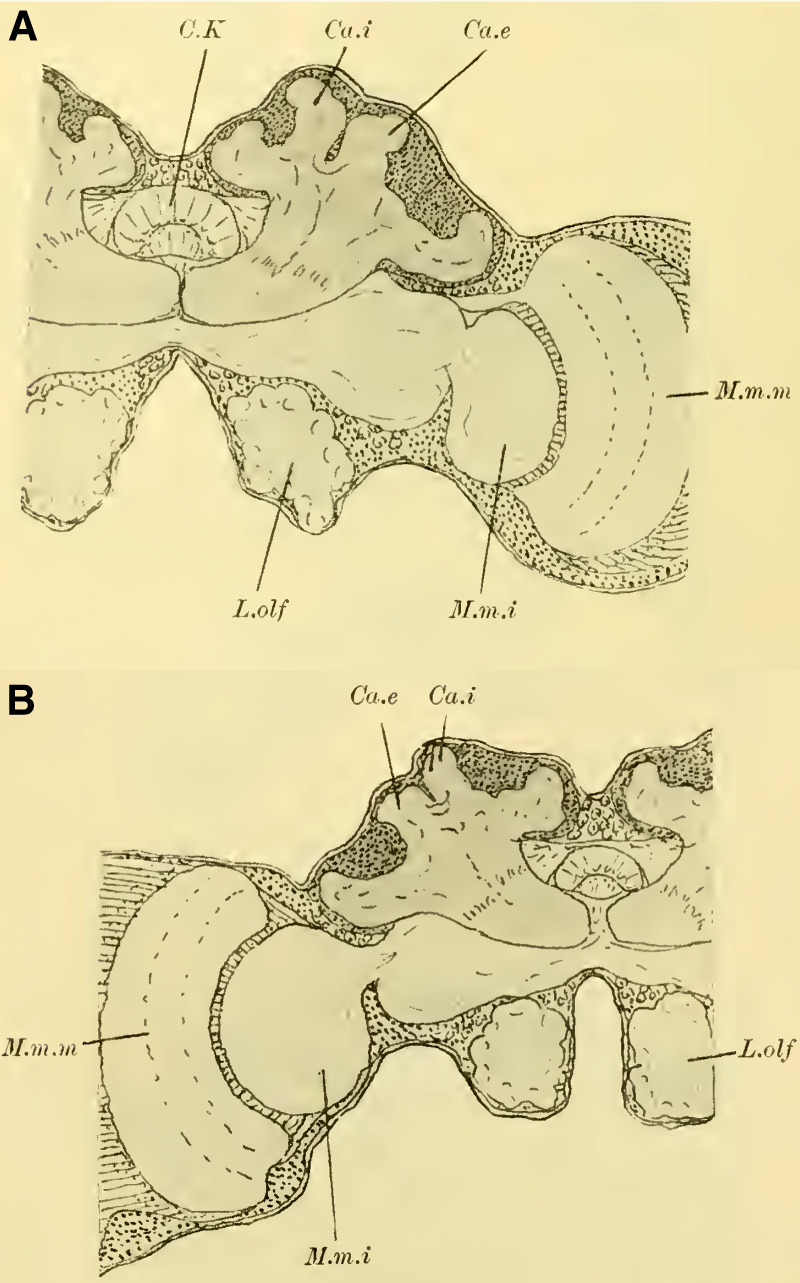
Two frontal sections through the brain of ***Andrena albicans*** ♀ (**A**) and ♂ (**B**). The labels are as usual. Scale: 1:60.

**TextFig. 21.**
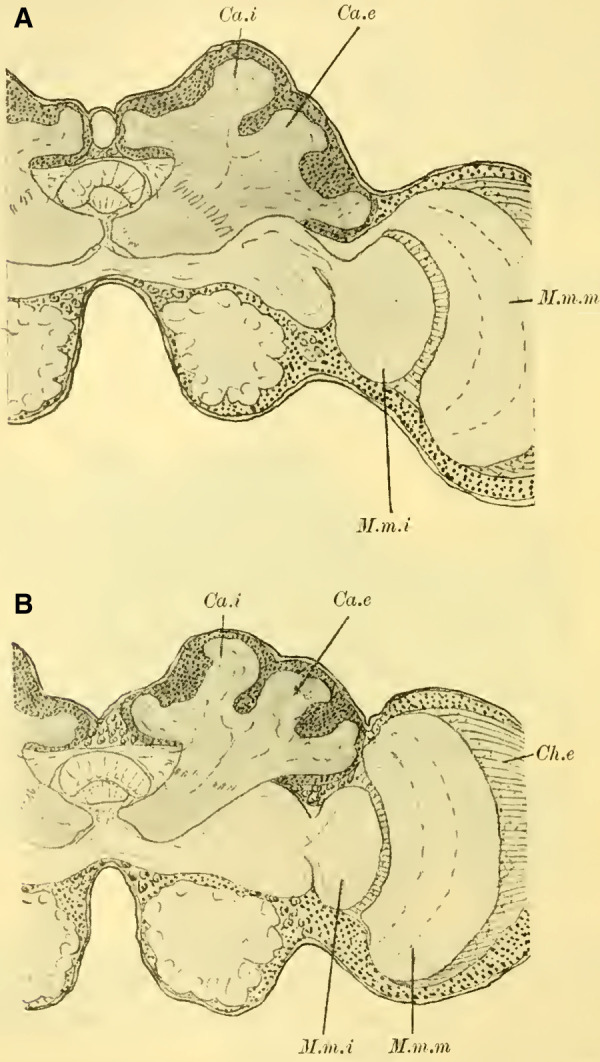
Two frontal sections through the brain of ***Eucera longicornis*** ♀ (A) and ♂ (B). The labels are as shown in TextFig. 12. Scale: 1:52.

It should be noted that in ***Andrena apicata*** (**TextFig. 22**) and in ***Eucera***, the trajectory of the sulcus is straighter from front to back in females compared to males, suggesting that this arrangement is likely to be found in most solitary apid bees.

**TextFig. 22.**
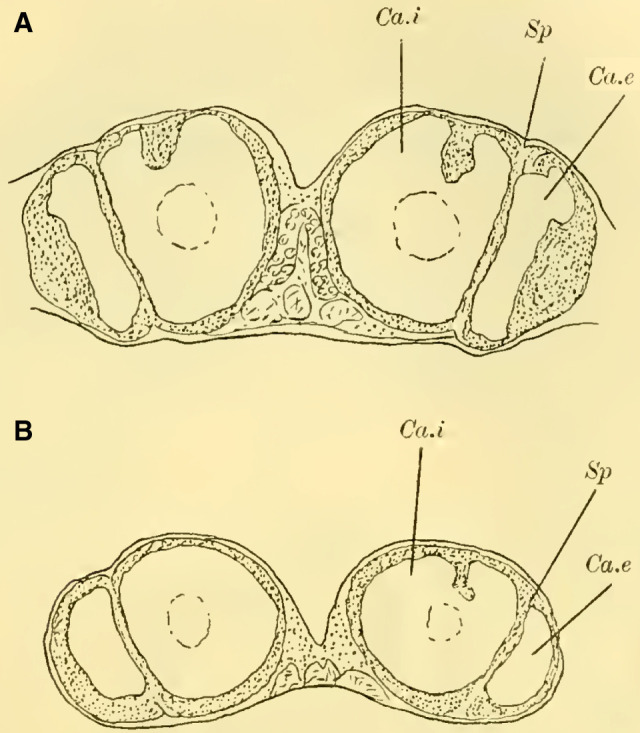
Two horizontal sections through the brain of ***Andrena apicata*** ♀ (A) and ♂ (B). Sp furrow. Scale: 1:60.

### c) Social Apidae

In bumblebees, the development of the mushroom body has made significant progress compared to solitary bees. The globuli have become taller than in ***Anthophora*** and extend laterally over the lobus opticus (**Fig. 29 on Plate 21**). As a result of this growth, the indices in bumblebees (**Table IV**) reach the highest values measured among the Apidae. These values, however, are not the same for all species. For example, in ***Bombus agrorum*** ♀ (♀ = queen), they are larger than in ***Bombus lapidarius*** ♀, and even larger, as indicated by the workers, in ***Bombus terrestris*** ♀.

Sexual differences are evident in the arrangement of the calyces in relation to each other (**TextFig. 23**), with the furrow (Sp) appearing straighter in the queen (**TextFig. 23A**) compared to the workers and males. (In Apis mellifica, this straight course is observed in workers, according to (Jonescu, 1909) and my findings.) Additionally, there are differences in the size ratios of the mushroom bodies (**TextFig. 24A-C**).

**TextFig. 23.**
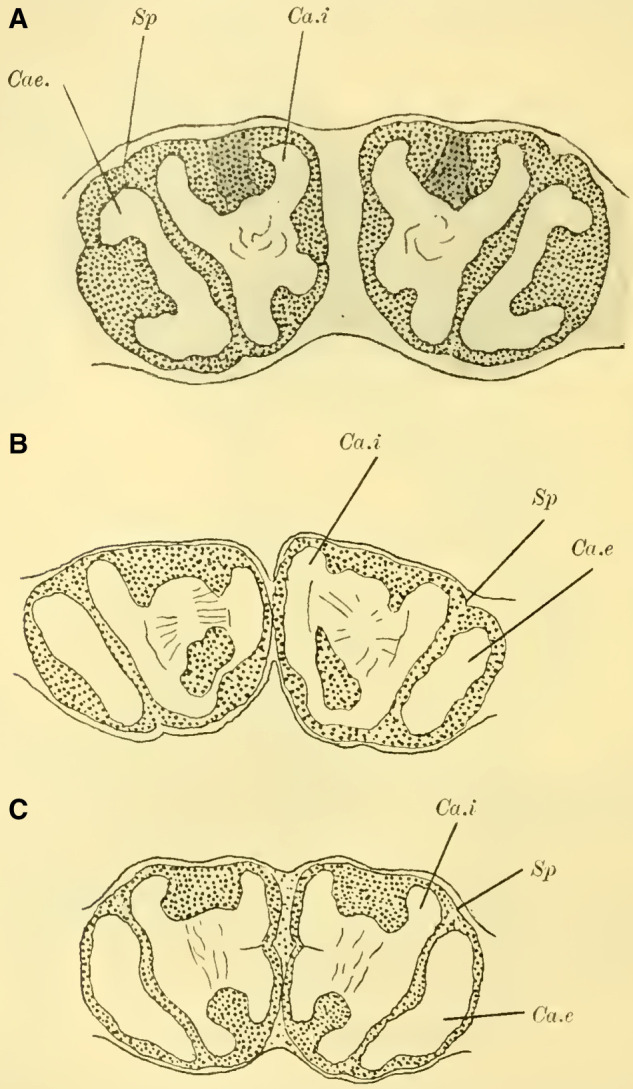
Three horizontal sections through the brain of ***Bombus agrorum*** ♀, queen (A), ☿, worker (B), ♂, male drone (C). Sp furrow. Scale: 1:40

**TextFig. 24:**
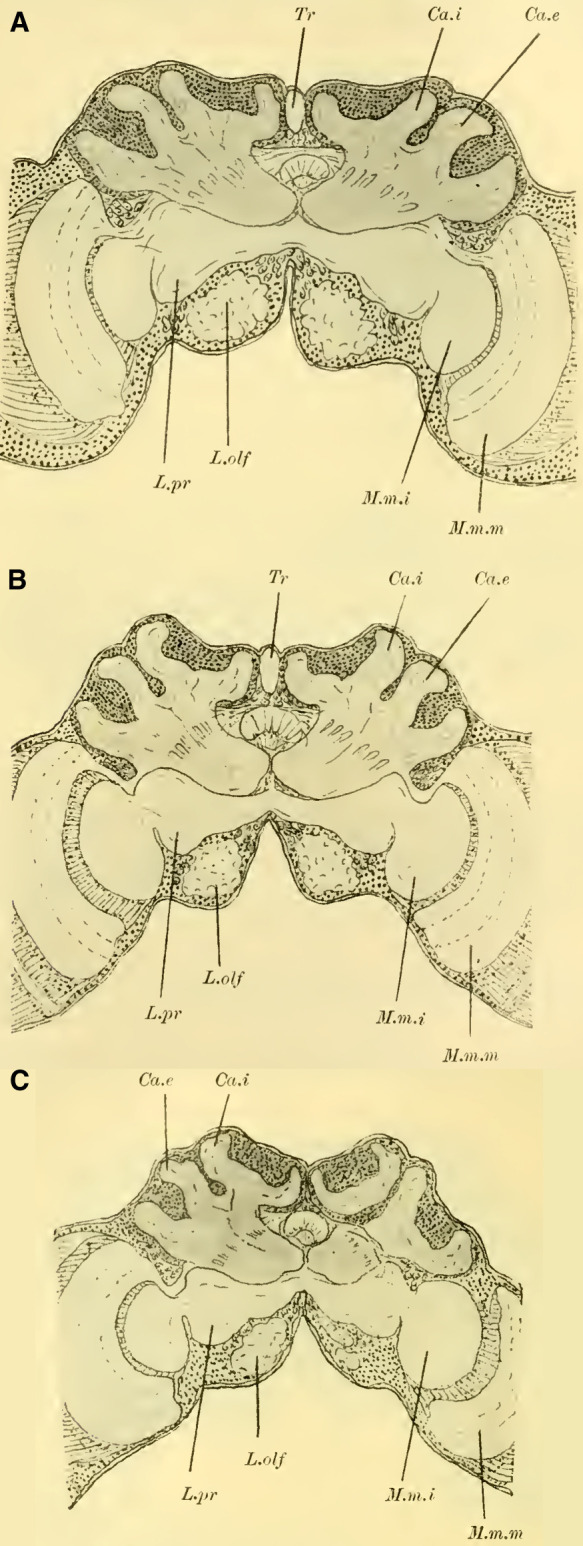
A-C. Three frontal sections through the brain of ***Bombus agrorum*** ♀, queen (A), ☿, worker (B), ♂, drone male (C). Labels as in **TextFig. 12**. Scale: 1:40

Examining the series of histological sections leads to the conclusion that the queens's brain is more highly developed than that of the worker, both in absolute and relative terms.

This conclusion is fully supported by the measurements. In both ***Bombus lapidarius*** and ***Bombus agrorum***, the queens surpass the workers in three indices, only in the a/c ratio (width of the mushroom bodies, greatest distance between the medullae) do they lag behind due to the greater extension of the optic lobes. (The theoretical significance of this fact will be discussed in the summary discussion). The males are the least well-developed, which is less evident in **Table IV** for ***Bombus lapidarius*** ♂ as the section's orientation slightly exaggerates the height of the mushroom bodies, but is more clearly expressed in ***Bombus terrestris***.

In ***Apis mellifica***, the order is different, as described in detail by (Jonescu, 1909), where the mushroom body of the worker bees are relatively the largest, followed by the queen females, and finally the drone males. (I already mentioned the different course of the furrow) I can confirm these results through my own research (see also **Table IV**). At first glance, the indices of ***Apis mellifica*** may appear small compared to those of bumblebees. However, this phenomenon becomes understandable when considering that the admired high organization of the bee colony primarily relies on a highly developed division of labor, which enables the colony as a whole to achieve great feats without requiring exceptionally high development of individual instincts. One could almost say the opposite. For example, compare the immensely versatile activities of a bumblebee female queen with the specialized reproductive instincts of an *Apis* queen. Additionally, it is possible that further division of labor has occurred among the worker bees, with some individuals being responsible for foraging while others focus solely on the propagation of the new generation. This would also provide an explanation for (Bachmetjev, 1909)’s findings of two polymorphic forms among the worker bees through his analytical-statistical investigations.

Differences in the arrangement of the optic neuropils on horizontal sections are also evident in the lobus opticus, particularly pronounced in bumblebees (**TextFig. 25**) and less so in ***Apis mellifica***. While the lobus opticus of females and queens does not exhibit any significant deviations from the typical structure, and the medulla may not be as strongly rotated around its vertical axis as in ***Xylocopa*** (**Fig. 18 on Plate 20**), this rotation is highly noticeable in males (**TextFig. 25B**, M.m.m). The reason for this is apparently a significant displacement of the retina towards the anterior surface of the brain in males. The lamina (M.m.e) has followed this movement, and the medulla has rotated in such a way that all fibers of the outer chiasma (Ch.e) can penetrate the anterior concave surface.

**TextFig. 25.**
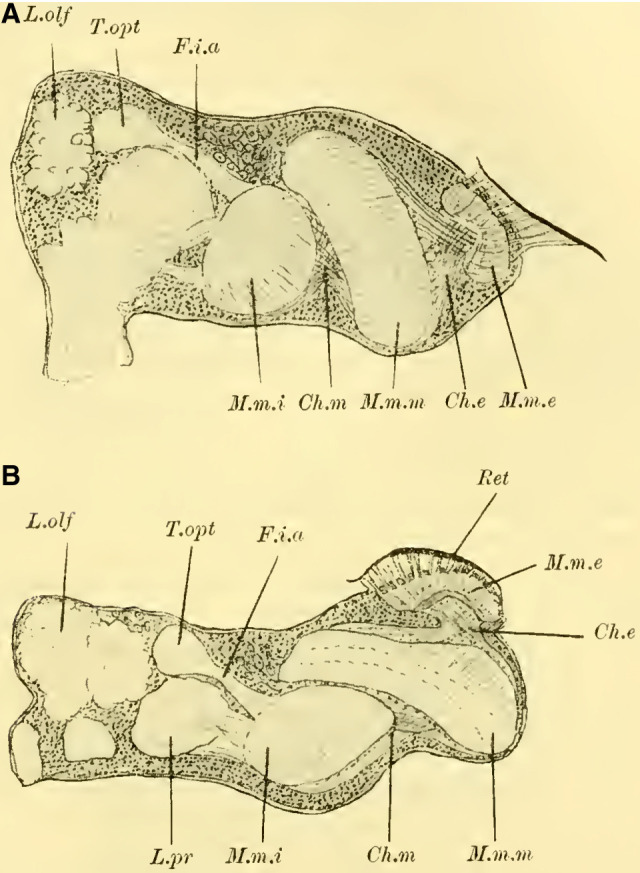
Horizontal sections through the brain of Bombus agrorum. **A** = Female type ♀ (queen) and ☿ (worker), **B** = ♂ male type. Ret Retina, M.m.e, M.m.m, M.m.i lamina, medulla, and lobula, Ch.c, Ch.m outer, middle chiasma, L.olf Olfactory lobe, L.pr Protocerebral lobe, T.opt optic tubercle, F.i.a Inferior anterior fascicle. 1:52

### d) Cuckoo bees (parasitic bees)

Examined: ***Nomada succincta/Psithyrus vestalis***

Their parasitic lifestyle has led to a series of modifications, particularly in their external morphology, such as the loss of the pollen-collecting apparatus and reduction of hair. They have also experienced a similar reduction in the development of their instincts, which is evident in a significant reduction of the mushroom body. In the case of ***Nomada succincta***, this fact is less easily observable due to the still unresolved question of its ancestry. However, in ***Psithyrus*** (**Figure 30 on Plate 21**), which is undoubtedly derived from ***Bombus***, the reduction of the mushroom bodies is evident.

In this species, although the optic lobes are well-developed, there is a decrease in the relative width and height of the mushroom body, as evident from the comparison of **Tables IV and V**. In comparison, the males appear to have lost only a small number of instincts (compare ***Psithyrus vestalis*** ♂ in **Table V** with ***Bombus terrestris*** ♂ in **Table IV**) and have maintained a similar level of brain development. This is similar to how the reduction in hair primarily affects females. (Friese, 1888) further states that the parasites compensate for sexual dimorphism, but in the case of these two mentioned species, dimorphism still exists in terms of the central nervous system. However, the regression of the mushroom body has reached such a degree in females of both ***Nomada*** and ***Psithyrus*** that it is now the males, rather than the females, who are far superior (**Table V, D**).

**Table V:** 

	a (µ)	b (µ)	c (µ)	h (µ)	a/b	a/c	h/b	h/c
Nomada succincta ♀	1258,6	998,2	2126,6	455,7	1,26	0,591	0,456	0,214
„ „ ♂	1063,3	824,6	1779,4	390,6	1,28	0,591	0,473	0,219
Psithyrus vestalis ♀	1475,6	1302,0	2517,2	672,7	1,13	0,586	0,516	0,267
„ „ ♂	1258,6	954,8	1996,4	542,5	1,31	0,630	0,568	0,271

	D a/b	D a/c	D h/b	D h/c
Nomada succincta ♀	}−0,02	0,000	−0,017	−0,005
„ „ ♂				
Psithyrus vestalis ♀	}−0,18	−0,044	−0,052	−0,004
„ „ ♂				

Labels as in Table I and II

Also, **TextFig. 26** reveals that in males (**B**), the mb globuli are well-developed relative to the rest of the brain, while in females (**A**), the size of the optic lobe is notable.

**TextFig. 26.**
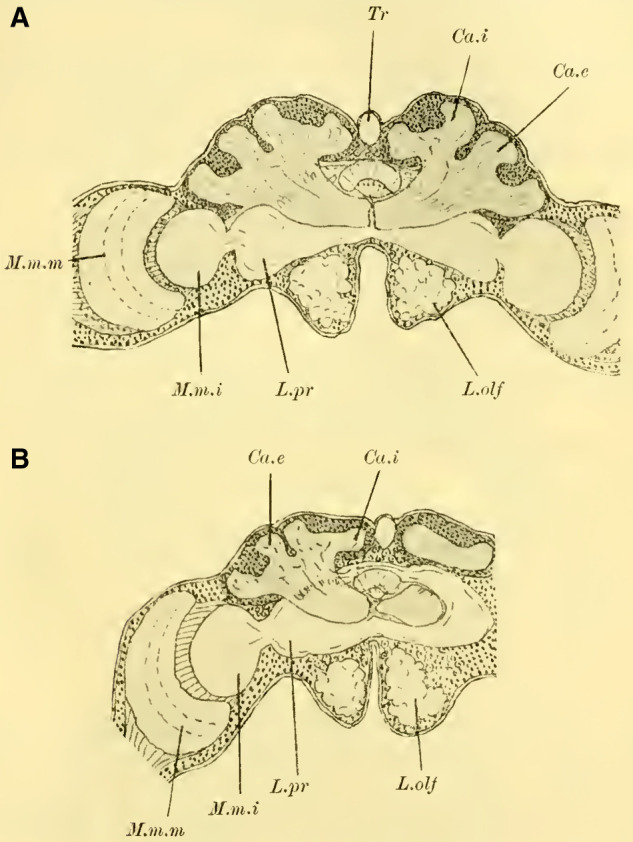
Two frontal sections through the brain of ***Nomada succincta.*** A = female (♀), B = male (♂). Labels as in TextFig. 12. Scale: 1:40.

These highly developed males suggest that the parasitic species must have been well-developed before transitioning to a parasitic lifestyle. The lobes of the optic and olfactory systems show no signs of rudimentary structures; in fact, they have likely further developed after the transition to parasitism. This is because these lobes are of utmost importance for the parasites to locate the nests of their hosts.

### F. Vespidae

Already in whole-mount preparations (**Fig. 6 on Plate 18**), it becomes apparent that we are dealing with highly developed forms in wasps, and at the same time, the shape and arrangement of the large calyces (Ca) suggest significant differences compared to bees. These differences are indeed significant in histological examinations to the extent that the first investigator, (Flögel, 1878), expressed the view that the brain of a wasp differs more from that of a bee than that of a bee from ***Blatta***. It was only (Viallanes, 1887a) who demonstrated the fundamental similarities in the structure of the brain between wasps and bees. His excellent findings leave little to add, so I will only briefly report on the relevant aspects in this case.

The mushroom bodies (**Fig. 31 P.K, on Plate 21**) have undergone extraordinary development upwards and laterally, and the two globuli are positioned almost directly next to each other, so that the furrow (**TextFig. 27** Sp) runs in an almost straight line in all three forms, perhaps slightly more obliquely in males (**C**) compared to queen females and workers. The neuropil of the calyces has extended forward and backward over the protocerebral lobes, exhibiting various swellings and thickenings to increase its surface area. Particularly noteworthy is a consistent indentation that runs all around the upper edge, giving it a double-lobed appearance on frontal sections (**Fig. 31 on Plate 21**).

**TextFig. 27.**
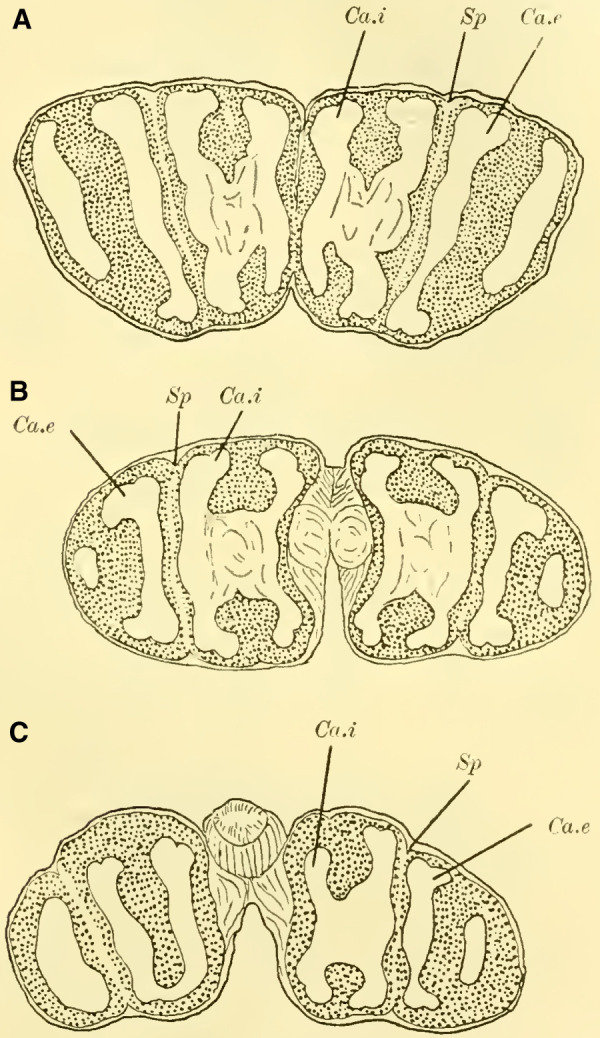
Three horizontal sections through the brain of Vespa vulgaris. A = female type, ♀, queen, B = ☿, worker, C = ♂, male. Labels as usual. 1:40**.**

The voluminous stalks (St) descend approximately vertically until reaching the height of the central body (C.K), tapering conically downwards. From there, the anterior root extends directly forward. It is notable not only for its extremely small diameter (see **Fig. 6** R.a, **on Plate 18**) but also for its structure, as it does not consist of nearly homogeneous neural tissue like in other Hymenoptera. Instead, it represents a fine bundle of fibrils with initially few embedded nuclei. As the anterior root approaches the frontal surface of the brain, the number of nuclei increases, while the fibers become less distinct and eventually disappear entirely between the frontal ganglionic cell layer of the brain.

The inner root (**Fig. 31,** R.i, **on Plate 21**), which (Flögel, 1878) completely overlooked, also consists of bundles of fine fibrils that curve downwards and merge with those of the opposite side. This direct connection of the inner roots is known only in wasps among Hymenoptera. Among other tracheates, direct connections between fibril bundles, referred to as “stalks,” with those of the opposite side are found only in very primitive forms. Therefore, it is undoubtedly a feature that was independently reacquired by wasps. Based on my investigations, it is clear, particularly from studies on pupae, that fibers directly penetrate (partially crossing) into the central body from these inner roots. This fact, which (Viallanes, 1882) could not determine definitively, appears to be beyond doubt, especially after conducting studies on pupae.

Among the different genera of wasps, there are variations in the size ratios of brain structures (**Table VI**), as well as between the sexes (**Table and TextFig. 28A–C**), following the same pattern observed in bumblebees. The female queen (A) stands out in both width and height development compared to other forms. The worker females **(B**) show a reduction in width rather than relative height of the mushroom bodies, while the male (**C**) is further disadvantaged and also experiences a decrease in relative height, although it surpasses the worker female in absolute brain size and development of the lobus opticus. **Table VI** provides numerical information about these relationships.

**TextFig. 28.**
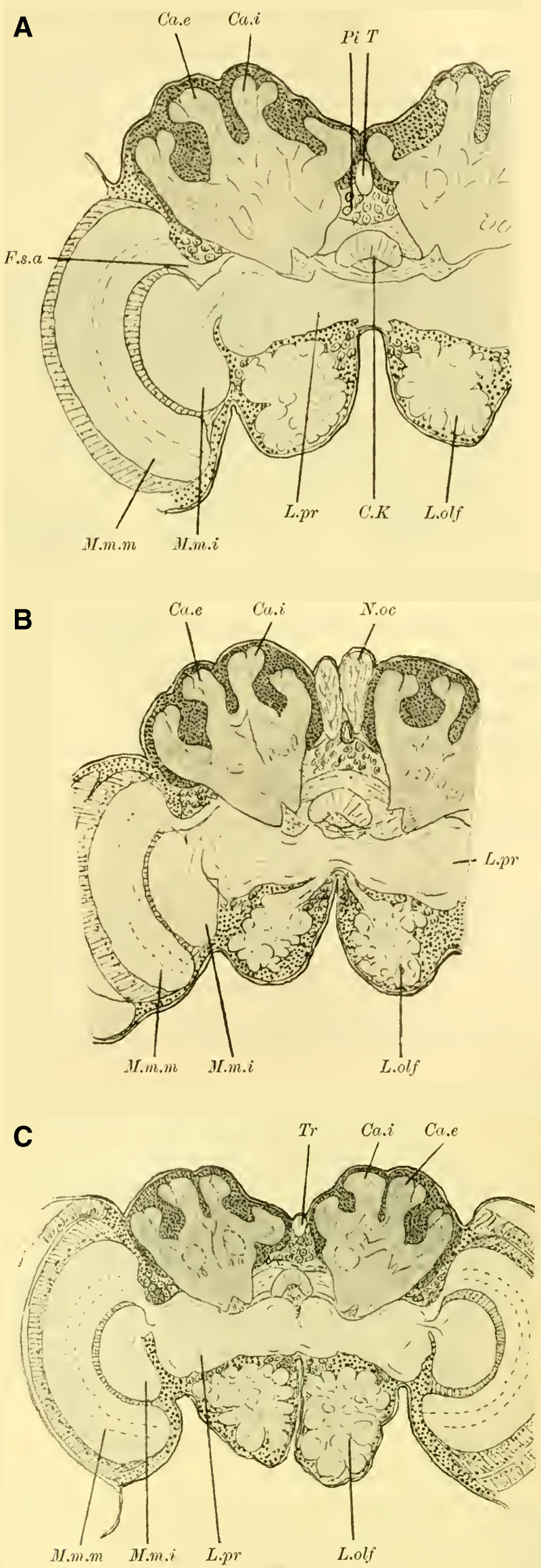
Three frontal sections through the brain of Vespa vulgaris. A =, ♀ queen, B = ☿ worker C = ♂ male. Tr Trachea, C.K. Central body, Pi Pars intercerebralis, F.s.a Superior anterior fascicle. Other labels see TextFig. 12. 1: 40.

**Table VI:** 

	a (µ)	b (µ)	c (µ)	h (µ)	a/b	a/c	h/b	h/c
Vespa cabro ♀	1996,4	1736,0	3385,2	824,6	1,15	0,589	0,475	0,243
Polistes gallica ☿	1258,6	954,8	1909,6	542,5	1,31	0,659	0,568	0,284
„ „ ♂	1302,0	1215,2	2256,8	542,5	1,07	0,576	0,446	0,240
Vespa vulgaris ♀	1736,0	1215,2	2300,2	716,1	1,42	0,754	0,589	0,311
„ „ ☿	1519,0	1215,2	1996,4	629,3	1,25	0,695	0,517	0,315
„ „ ♂	1345,4	1085,0	2126,6	564,2	1,24	0,632	0,520	0,265

### Summary and theoretical implications of the research findings

Despite the overall consistent brain structure of Hymenoptera, there are various differences observed among the different suborders, particularly in three variable parts: the mushroom body, the optic lobe, and the olfactory lobe.

The mushroom bodies are paired structures of the cerebrum located on each side of the midline, separated by the “sillon cérébral médian” (Viallanes, 1882). Initially, they are less prominent in species such as Tenthredinidae but progressively increase in size, expanding in height and width in groups like Uroceridae, Ichneumonidae, and Apidae, reaching their highest development in wasps (**Fig. 1-6 on Plate 18**). Within each mushroom body, two distinct globuli can be identified, consisting of characteristic ganglion cells and neuropil, separated by a furrow (la scissure du corps pédonculé) (Viallanes, 1882). This furrow, which only becomes clearly recognizable from the Ichneumonidae, runs only in the Cynipidae from posterior laterally to anterior medial, in most other species from posterior medial to anterior lateral—quite extreme in the Ichneumonidae and Braconidae—and only rarely in highly developed forms (***Apis mellifica***, ***Vespa vulgaris*** ♀, approximately straight from front to back.

The ganglion cells within the globuli of Aculeata undergo further differentiation, with a distinct group of highly stainable medial cells surrounded by a ring of a second group of cells. Initially, they are positioned far apart from their counterparts on the opposite side, allowing the protoplasm-rich ganglion cells of the pars intercerebralis to extend extensively. However, as development progresses, they grow toward the midline, encroaching upon the cells of the Pars intercerebralis, which becomes more restricted in number and size. Eventually, the inner globuli of both sides come into contact in the midline. Lateral growth is also evident as the calyces, initially sitting on the protocerebral lobes, extend beyond their lateral edges, reaching the inner and potentially middle neuropils of the optic lobe.

The calycal neuropil of the globuli exhibits various types, with four distinguished categories. The simplest form, found in Tenthredinidae, is the club-shaped or piston type. This develops into the shell type seen in Cynipidae and Uroceridae through a medial deepening. As the edges of the shell continue to grow and cause an increase in surface area through manifold thickenings and bulges, the chalice type of Ichneumonidae and Braconidae arises on the one hand, and the cup type of the Aculeates on the other.

The optic lobe in all investigated Hymenoptera consists of the intersecting fibers and neuropil masses described in detail for Tenthredinidae. However, the shape and orientation of these optical neuropils may vary among the suborders. Notably, the medulla and lobula, which are nearly perpendicular to the length of the optic lobe in Tenthredinidae, show a tendency to rotate around their vertical axes, with medulla facing forward with its concavity and the lobula facing backward, most prominently observed in Ichneumonidae.

In these suborders, the olfactory lobe, whose size is variable, reaches its highest qualitative and quantitative development.

The question now arises as to whether these results can be utilized for phylogenetic considerations.

Discussions about the phylogenetic position of Hymenopteran families have primarily relied on general biological considerations and the different development of mouthparts. However, the latter seem to be highly variable organs and should be used with caution for phylogenetic speculations. According to (Demoll, 1908, 1909), for example, the Uroceridae (Siricidae) possess more primitive mouthparts compared to the sawflies, while they rank higher in terms of central nervous system development. I also refer to the previously discussed convergent phenomena observed in species living under similar nutritional conditions but arising from completely different basic forms. (Compare also the work of (Langhoffer, 1898) on the mouthparts of solitary bees, which leads to unacceptable consequences.)

A more fruitful approach, in my opinion, would be to consider the varying levels of instinct and reflex activity observed in life history, such as in behavior, egg-laying, nest-building, parental care, etc. This approach aims to eliminate subjective elements and potential anthropomorphizations and instead focuses on the development of the material basis of these instincts, namely the brains, and, in turn, the diverse development of the mushroom body as the main centers for reflexes and associations.

From this perspective, the sawflies (Tenthredinidae) are undoubtedly considered the most primitive among the examined Hymenoptera. The conditions observed in the gall wasps (Cynipidae) and horntails (Uroceridae = Siricidae) suggest a close relationship with the sawflies. In both groups, the club-shaped type of the mushroom bodies has further developed into a shell-shaped form, albeit in different ways. In the gall wasps, the inner globuli shift backward and the outer globuli shift forward, resulting in the furrow running from the posterior lateral to the anterior medial region, opposite to what is observed in all other examined species, including the horntails. The development of the mushroom bodies in gall wasps is more pronounced than in horntails, with the ganglionic masses overgrowing the protocerebral lobes and the associated calycal neuropil and mb stalks exhibiting relatively greater expansion and thickness.

However, the gall wasps (Cynipidae) cannot be placed in the same line as the horntails (Uroceridae) because, despite their higher development of the globuli, the neuropils of the lobus opticus still retain their original position seen in sawflies. Both groups must have evolved separately from the sawflies, with the gall wasps further developing the club-shaped type of the globuli into the shell-shaped type and acquiring the unique displacement of the furrow, while retaining the position of the neuropil of the lobus opticus. On the other hand, the horntails (Uroceridae) evolved a less developed shell-shaped type of globuli, maintaining the position of the furrow as observed in all other Hymenoptera. However, the neuropils of the lobus opticus undergo the rotation that is common to almost all Hymenoptera and is further developed in the Ichneumonidae.

Therefore, there are more indications to suggest that the further development did not occur in the gall wasps (Cynipidae) but rather in the horntails (Uroceridae), as the volume of the calycal shell is increased by the expansion of the edges, and the globuli reach their next stage of development in the ichneumon wasps. However, before the Ichneumonidae fully developed the chalice-shaped form of the globuli, the extreme arrangement of them in a row, and the rotation of the neuropil of the lobus opticus, the fossorial wasps (digging wasps, sphecoid wasps, e.g., Sphecidae, (TN: today they belong to the Apoidea), the ancestral group of aculeate wasps, must have diverged from this lineage. In these fossorial wasps, the fully developed edges of the shell turned inward, leading to the formation of the cup-shaped type characteristic of aculeate wasps. A similar conclusion was reached by (Verhoeff, 1892) based on purely biological considerations. According to him, the digging wasps evolved “from the lineage of ichneumon wasps,” and “the first step in the divergence of the fossorial lineage was that the mother wasp did not simply lay each individual egg on the prey but buried it together with the prey in an underground chamber and claimed it completely for itself.” I believe that this interpretation can be further clarified by stating that this divergence from the lineage of ichneumon wasps did not occur in currently living species but must have taken place earlier, as discussed above.

The wasps themselves exhibit such aberrant brain structures that their derivation from currently living fossorial wasps (Sphecidae**)** is also no longer plausible; they must have already branched off from ancestral forms of the present species, from “pro-fossorial” wasps. According to (Verhoeff, 1892), they are descended from “Pro-trypoxylidae,” forms that were closest to the present-day Trypoxylidae (Crabronidae) with the inclusion of eumenid wasps (Potter wasps, Eumeninae, Vespidae). Unfortunately, I was unable to examine the eumenid wasps, but they may potentially reveal interesting intermediate forms.

The brain of present-day fossorial wasps is already built entirely according to the Apis type. Therefore, a transition to the archiapids occurs naturally from them (cf. (Müller, 1872), from which a progressively relative growth of the mushroom bodies and a gradually more distinct sexual dimorphism favoring females can be demonstrated. This is evident both in the Gastrilegidae (highest form: ***Anthidium***) and in the Podilegidae, largely following an order that corresponds to the one established by (Friese, 1891), with a primary focus on the development of the collection apparatus. An even greater development of the mushroom bodies is found in the Podilegidae, which transition from ***Apis mellifica*** to ***Anthophora*** and ultimately to ***Bombus***, encompassing their highest forms.

Thus, by primarily considering the different development of the central nervous system, in part in accordance with results obtained from general biological considerations and based on the development of the collection apparatus, a table of relationships for the suborders and families of Hymenoptera would result in approximately the following arrangement (TextFig. 29).

**TextFig. 29.**
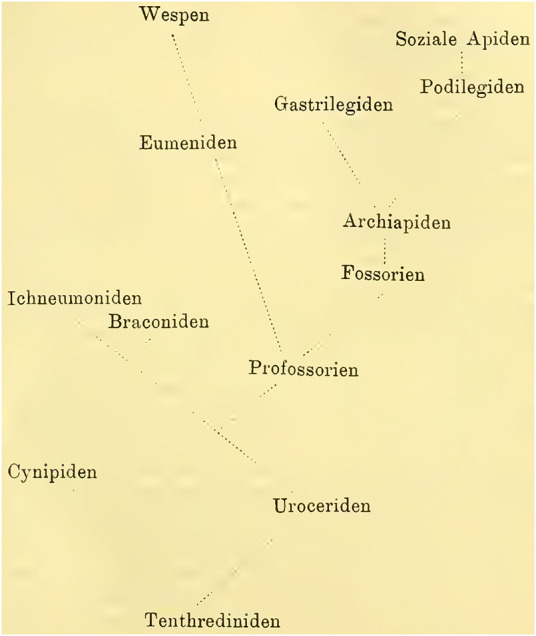
A hypothetical phylogenetic tree based on neural characters of mushroom body features in diverse hymenopteran groups.

Regarding the phylogenetic position of individual genera of solitary Apidae among each other, my measurements have not revealed anything substantially new. The results obtained align mainly with the relationship tables established by (Friese, 1891).

Furthermore, it was found that variations in the development of the central nervous system also exist among solitary Apidae. In accordance with Friese's phylogenetic tree (Friese, 1888, 1891), there is a tendency for increasing width and height of the mushroom bodies, which can be nicely demonstrated in the series of Gastriligidae from Osmia to Chalicodoma, Megachile, and Anthidium.

Additionally, the highest values are reached in the series of Podilegidae (*Bombus*), which, however, experience a reduction during the transition to perennial states (*Apis*) (cf. the section “Social Apidae”). Finally, in response to the initial question raised, it was found that sexual differences exist in all studied species of solitary Apidae, although in lower forms, one cannot yet speak of a predominance of the female sex. For example, in these species, a greater width of the mushroom bodies in females can be compensated by a greater height in males (***Colletes cunnicularius***, ***Eriades crenulatus***), and vice versa (***Halictus calceatus***, ***Andrena albicans***). Similarly, this can also occur within two species of the same genus (***Andrena fulva and albicans***). The predominance of the female sex in terms of the development of mushroom bodies, which has differentiated throughout phylogenesis, becomes increasingly evident only in the more highly evolved species.

Furthermore, it has been observed that male parasitic bees show only a minor reduction, while significant regression of the mushroom body can be observed in females. However, the optic lobes and olfactory lobes remain well-developed in females.

Finally, I would like to point out that in non-perennial bumblebee and wasp colonies, the female (queen) is the most highly developed, followed by the female workers and ultimately the males, whereas in ***Apis mellifica***, the female workers are higher than the females (queen) and males (drone). This result seems to be of some interest, as some have argued, particularly (Buttel-Reepen, 1915), that bumblebee and wasp workers are merely small, poorly nourished, but otherwise morphologically and anatomically complete females, and therefore cannot be directly compared to the female workers of ***Apis mellifica***. In contrast, I believe that bumblebee female workers differ from the female queen not only in absolute but also in relatively lower development of the mushroom bodies and thus the instincts. Therefore, we must assume that even in bumblebee and wasp eggs, just as (Weismann, 1900, 1904) suggested for honeybee eggs, there are three separate primordia for the three different forms.

Undoubtedly, ***Apis mellifica*** has also gone through a bumblebee-like state. It was only when the colonies became perennial that there was a gradual regression and atrophy of most female instincts, except for the sexual ones; this regression probably even proved advantageous for the colony and thus had selective value. On the other hand, the workers, initially regressed compared to the females (queen), still possessed many of the same instincts and even developed some additional ones, making them superior to the queens in a secondary sense (cf. also the discussions by (Buttel-Reepen, 1900), l.c. p. 49 ff.). In this regard, it might be interesting to compare the development of mushroom bodies in Corsican bumblebee species (***Bombus xanthopus***, among others), which according to (Ferton, 1905) (25) and (Buttel-Reepen, 1915) are likely to be perennial or at least in the process of becoming so, or in the Nordic ***Bombus kirbyellus*** and ***hyperboreus***, which (Schneider, 1894, 1909) found to have returned to solitary behavior in the vicinity of Tromsø, with the findings in native species.

A more detailed investigation of postembryonic development might yield further valuable results. Until recently, only minor histological changes were assumed, until (Bauer, 1904) noted that “there is hardly an organ system in insects that undergoes such extensive metamorphosis of its parts as the nervous central organ.” However, Bauer's research is primarily histological and provides limited information on changes in the shape, size, and position of individual brain parts. (Jonescu, 1909) found a shell-like shape of the calyces in ***Apis mellifica*** pupae, which slowly develops into a cup shape. I can confirm this finding, also in pupae of ***Vespa vulgaris***. Furthermore, I observed that the anterior root, which in the adult insect is only a fine fibril strand, is much more voluminous in the pupa and in terms of size and shape strongly resembles the anterior root of the Apidae.

Therefore, it is not unlikely that the different types of central nervous systems appearing in phylogenetic development can also be traced in ontogeny, and that reductions, such as those found in parasites and Apis mellifica, may become apparent during postembryonic development.

**Freiburg i. B., April 1910** **(original German publication)**

**Jena, 2023** **(translation to English)**

## Footnotes

1 Francis Delafield was an U.S. physician and pathologist, 1841-1915

2 I refer to the paired ganglion cell clusters with their respective neuropil in higher Apidae on each side as globules (‘globuli’), two of which on each side form a mushroom body

3 sodium hypochlorite

4 Newton coined the term calix in his paper on the cockroach brain in 1879

## Supplementary note

Supplemental Material is available for this article. Please note that the term “Figures” (on Plates 18–21) in the text refers to Supplemental Figures (S1), while the term “TextFig.” refers to Figures included in the main article.
